# Cell Painting PLUS: An Iterative Staining‐Elution Protocol for High‐Content Phenotypic Screenings

**DOI:** 10.1002/cpz1.70368

**Published:** 2026-04-27

**Authors:** Marlene Wedler, Elena von Coburg, Jose M. Muino, Lars Valentin, Nils Körber, Sebastian Dunst, Shu Liu

**Affiliations:** ^1^ German Centre for the Protection of Laboratory Animals (Bf3R) and Experimental Toxicology German Federal Institute for Risk Assessment (BfR) Berlin Germany; ^2^ Institute of Biology Freie Universität Berlin Berlin Germany; ^3^ Innovation Centre Food Chain Modelling and Artificial Intelligence, Department Information Technology German Federal Institute for Risk Assessment (BfR) Berlin Germany; ^4^ Centre for Artificial Intelligence in Public Health Research Robert Koch Institute Berlin Germany; ^5^ Equal contribution

**Keywords:** cell‐based assays, Cell Painting PLUS (CPP), high‐throughput high‐content screenings, morphological profiling, new approach methodologies (NAMs)

## Abstract

Cell Painting (CP) methods use a combination of fluorescent dyes to label multiple cellular compartments simultaneously, enabling the comprehensive analysis of phenotypic changes through morphological profiling. Here, we present a detailed protocol for the Cell Painting PLUS (CPP) method along with an automated image and data analysis strategy. In CPP, the iterative staining, elution, and re‐staining of cells with seven fluorescent dyes enable multiplexed analysis of nine cellular compartments and organelles. Each dye is captured in individual imaging channels to specifically distinguish effects on the plasma membrane, actin cytoskeleton, cytoplasmic and nucleolar RNA, lysosomes, nuclear DNA, endoplasmic reticulum, mitochondria, and Golgi apparatus. During the image analysis procedure, 894 morphological features (readouts) are extracted from single cells to generate comprehensive phenotypic profiles that resemble morphological perturbations. For efficient processing of the extracted features, we provide an automated data analysis workflow, which includes quality control, data normalization, and various data visualization tools. This workflow is based on a customized *CPPAnalyzer* Jupyter notebook and a *CPPManager* KNIME workflow, which can be easily applied without any special bioinformatics knowledge. In this way, CPP expands the multiplexing capacity, customizability, and, importantly, organelle specificity of the available CP‐based screening methods. © 2026 The Author(s). *Current Protocols* published by Wiley Periodicals LLC.

**Basic Protocol 1**: Seeding of U2OS, MCF‐7, or HepG‐2 cells in multiwell plates and treatment of cells with compounds

**Support Protocol 1**: Preparation of assay‐ready compound plate

**Alternate Protocol**: Seeding and differentiation of primary RPTEC‐TERT1 cells in multiwell plates

**Basic Protocol 2**: Staining and imaging of cells using Cell Painting PLUS

**Basic Protocol 3**: Image analysis

**Basic Protocol 4**: Data normalization and data quality control

**Basic Protocol 5**: Visualization of data using *CPPManager* KNIME workflow

## INTRODUCTION

**Figure 1:**
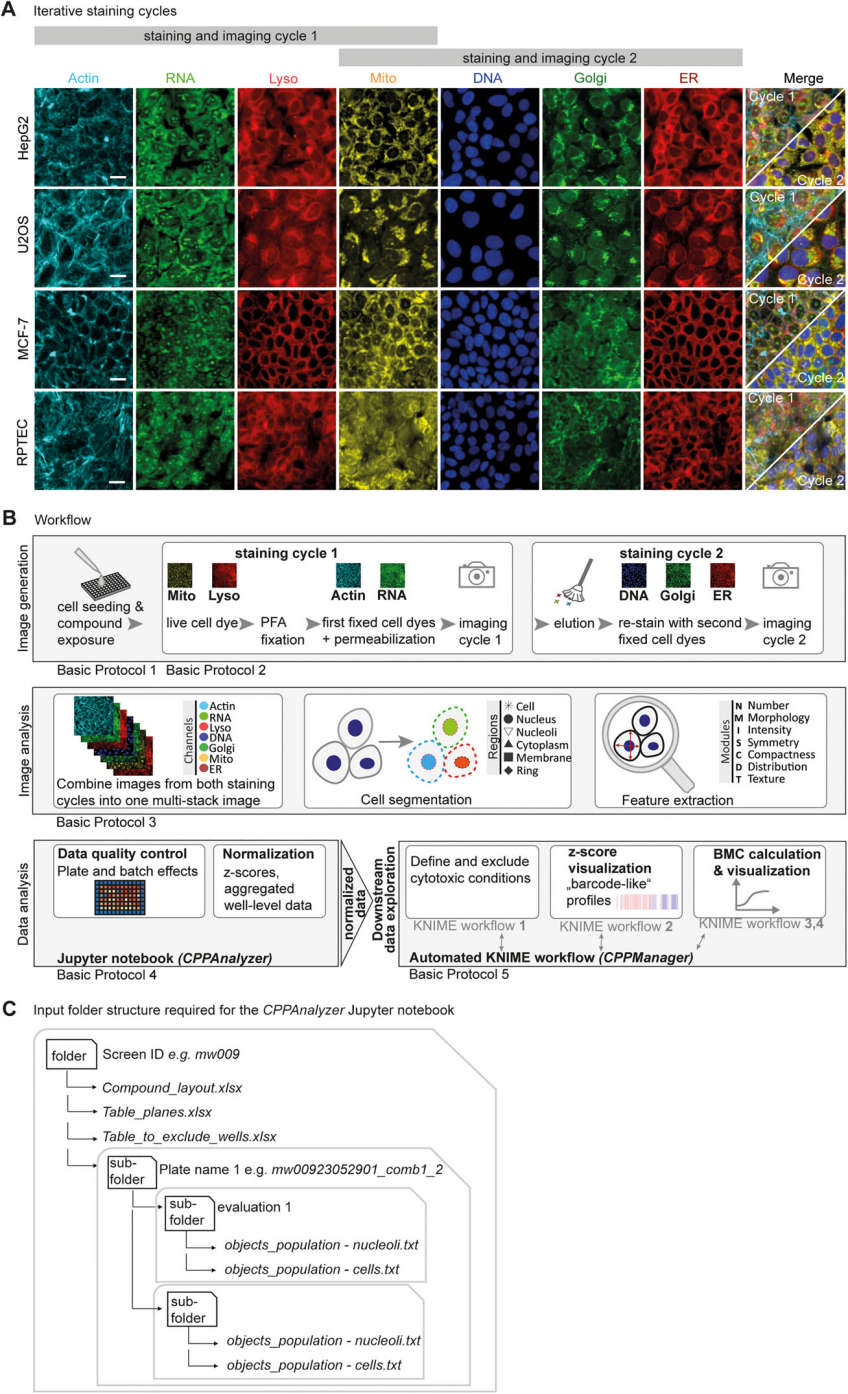
(**A**) The iterative staining cycles of Cell Painting PLUS were applied to four human cell lines: HepG2, U2OS, MCF‐7, and RPTEC. In cycle 1, actin, RNA (including cytoplasmic RNA and nucleoli), lysosomes, and mitochondria are stained and imaged. After elution, DNA, Golgi apparatus, and endoplasmic reticulum (ER) are stained. The mitochondrial dye is not eluted and is therefore present in both cycles 1 and 2. Scale bars, 20 µm. Actin, imaging channel with stained actin cytoskeleton; RNA, imaging channel with stained cytoplasmic and nucleolar ribonucleic acid; Lyso, imaging channel with stained lysosomes; Mito, imaging channel with stained mitochondria; DNA, imaging channel with stained deoxyribonucleic acid; Golgi, imaging channel with stained Golgi apparatus; ER, imaging channel with stained endoplasmic reticulum. (**B**) The workflow of the Cell Painting PLUS protocol presented here comprises an experimental procedure including staining, imaging, and downstream image and data analysis. Cells are first seeded in microwell plates and exposed to compounds of interest, and then subjected to the staining procedure. In staining cycle 1, the live‐cell dyes for Lyso and Mito are applied to cells. The cells are then fixed with PFA, followed by Actin and RNA staining. After imaging cycle 1, dyes (except Mito dye) are eluted from cells, and then the same cells are re‐stained with DNA, Golgi, and ER dyes and finally re‐imaged in cycle 2. During image analysis, images from both staining cycles are first combined into one multichannel image. Then, different cell regions are segmented and morphological features are extracted. Extracted features are subjected to data analysis using the *CPPAnalyzer* Jupyter notebook for data QC and normalization. Normalized data can then be fed into the downstream *CPPManager* KNIME workflow that calls different KNIME workflows for automated data visualization (cytotoxicity and phenotypic profiles), as well as, if applicable, BMC modeling and visualization. ER, endoplasmic reticulum; PFA, paraformaldehyde; BMC, benchmark concentration. (**C**) Required folder structure for the *CPPAnalyzer* Jupyter notebook. The main directory contains the following Excel files: Compound_layout.xlsx defines the treatment conditions assigned to each well. Table_planes.xlsx specifies, for each plate, the *z*‐plane that should be analyzed. Using the Table_to_exclude_wells.xlsx file, wells with known experimental errors can be excluded from the analysis (see Basic Protocol 4, steps 2‐4). The exported Harmony image analysis result files (in .txt format; see Basic Protocol 3, step 13) for each Harmony evaluation are stored in separate subfolders. If multiple Harmony evaluations were performed for a single plate (e.g., when evaluation 1 includes only part of the wells and evaluation 2 covers the remainder), this structure ensures that data from interrupted or restarted evaluations are correctly organized across separate evaluation folders. If only one Harmony evaluation (evaluation 1) was conducted, simply include only one evaluation folder.

High‐throughput/high‐content (HT/HC) phenotypic screening uses images of cells to evaluate phenotypic changes in cell morphology, for example in response to compound treatment or genetic perturbation (Chandrasekaran et al., 2024). It is essentially based on the assumption that morphological changes are a sign of the perturbation of cell functions and that similar morphological changes are associated with similar modes of action (MoAs). Cell Painting (CP) is a widely used *in vitro* phenotypic screening method that labels different cellular compartments using fluorescent dyes. The CP protocol originally published uses a defined set of dyes and captures stained cellular compartments and organelles in four or five microscopic channels (Bray et al., 2016). Consequently, signals from two fluorescent dyes are typically merged into the same imaging channel—e.g., Actin+Golgi and/or ER+RNA dyes—to maximize throughput capacity while maintaining high data density (Cimini et al., 2023; Nyffeler et al., 2020). However, this trade‐off of merged dye signals also limits the organelle specificity and diversity of CP data. To overcome this boundary, we recently published the Cell Painting PLUS (CPP) method, a further developed version of the original CP method (von Coburg et al., 2025a). CPP provides a novel iterative staining‐elution cycle that enables each dye to be imaged in a separate microscopic channel, thereby expanding the capabilities of CP‐based methods, especially where the number of microscopic channels is limited. The current version of CPP includes two staining cycles, in which seven fluorescent dyes are used to visualize actin cytoskeleton (Actin), cytoplasmic and nucleolar ribonucleic acid (RNA), lysosomes (Lyso), mitochondria (Mito), nucleus (DNA), Golgi apparatus (Golgi), and the endoplasmic reticulum (ER) (Fig. 1A).

CPP represents a valuable extension of the standard CP approach, which, depending on the scientific context, justifies the additional time required for the staining‐elution cycle. The basic costs of CPP and CP are comparable per single dye used. The additional reagent costs of CPP are due to the lysosome dye currently used and may decrease in future if alternative options for lysosome staining become available. Furthermore, depending on the scientific context and experimental demands, CPP provides high flexibility and customization options in regard to the selection of relevant dyes for cellular target compartments and organelles, enabling, for example, the inclusion of cellular structures such as lysosomes that are not covered by traditional CP‐based approaches.

The current version of the CPP protocol, presented here, allows cells to be stained with seven different fluorescent dyes (Fig. 1A). A first set of dyes (Mito, Lyso, Actin, RNA) is used in the first staining cycle (staining cycle 1) and imaged, and then the dyes are eluted to allow a second staining cycle (staining cycle 2) using different dyes (DNA, Golgi, ER), followed by a second imaging step (Fig. 1B). Importantly, each dye is imaged in a separate microscopic channel. Notably, the elution buffer was designed to avoid eluting the Mito staining. Therefore, the Mito signal is present in both imaging cycles 1 and 2 and serves as a reference for combining the images from the two staining cycles into a single multichannel image. In the subsequent image analysis procedure, a predefined set of 894 morphological features is extracted from single cells of staining cycles 1 and 2 images.

For data analysis, we built an automated workflow using a Jupyter notebook (*CPPAnalyzer*) that uses customized R code for quality control (QC) and normalization of numerical feature data. This Jupyter notebook provides a user interface that makes it possible to easily run the R code without the need of specific bioinformatics programming knowledge. The normalized data can then be explored using an automated KNIME manager workflow *(CPPManager)*. The Konstanz Information Miner (KNIME; Berthold et al., 2007) is an open‐source platform that uses visual programming elements (“nodes”) but also allows the integration of code snippets from different programming languages, such as R, Java, or Python (Berthold et al., 2007; KNIME AG, 2026). KNIME is operated using visual programming elements and can also be run by users with little or no programming experience. Thus, the data analysis workflow presented here, which is based on open‐source software tools (Jupyter, R, and KNIME), facilitates fast and easy processing of CPP data.

Here, we present a step‐by‐step guide for the versatile application of the CPP method to various cell lines. First, we describe the seeding of cells in multiwell plates, as well as compound treatment, for U2OS (bone, osteosarcoma), HepG2 (liver, hepatoblastoma), and MCF‐7/vBOS (breast, adenocarcinoma) cells (Basic Protocol 1), as well as for differentiated RPTEC‐TERT1 (kidney, hTERT‐immortalized) cells (Alternate Protocol). We then guide the reader through the staining and imaging protocol to collect CPP images (Basic Protocol 2) and further describe the image analysis procedure (Basic Protocol 3). Subsequently, we introduce a fully automated, customized CPP data analysis workflow using Jupyter notebook (*CPPAnalyzer*) for data normalization and QC (Basic Protocol 4). Finally, Basic Protocol 5 demonstrates how the normalized CPP data can be explored with the open‐source software KNIME (*CPPManager*), implementing data visualization tools, as well as optional benchmark concentration (BMC) modeling for multi‐concentration approaches.

## STRATEGIC PLANNING

### Cell Types and Cell Cultivation

The CPP protocol has already been applied to various human primary and cancer cell lines, including U2OS, HepG2, RPTEC‐TERT1, and MCF‐7/vBOS (von Coburg et al., 2025a). Although these cell models differ in terms of specific cultivation and cell seeding conditions, the CPP staining protocol proved to be universally applicable to all the tested cell lines. Notably, not only cells that form monolayers can be imaged; cells growing in a more complex, multilayer structure (such as RPTEC) were also successfully stained and imaged, demonstrating the high versatility of CPP.

Free‐floating cell suspensions are generally not suitable for CPP. Instead, cells need to be adherent and immobile during the staining and imaging procedures so that the same cells can be accurately registered and exactly matched from staining cycles 1 and 2.

If using a new cell type, we recommend testing the plates and seeding conditions beforehand. Each cell line requires optimized cell seeding conditions in order to produce reliable and reproducible phenotypic profiles. CP is traditionally applied to subconfluent cells at densities ranging from 400 to 2,000 cells per well (Gustafsdottir et al., 2013; Nyffeler et al., 2020; Wolff et al., 2025) in order to avoid overlapping cell growth that might impair single cell analysis during image analysis. However, subconfluent cells might not reflect the physiological conditions of the complex microenvironment found in tissues in regard to cell‐to‐cell contact and cell proliferation stage. Thus, the choice of cell confluency level requires a trade‐off between optimal imaging conditions and the representation of physiologically relevant cell conditions. Importantly, apart from the cell confluency, the number of cells imaged and analyzed needs to be considered for robustness of the assay: low cell confluency might require the capture of more fields per well to ensure that sufficient cell numbers are analyzed for statistically robust results. Thus, instead of giving recommendations for definitive cell seeding numbers, we encourage users of these protocols to test and adjust the cell number according to their specific experimental needs. If a more physiological setup is preferred, addressing specific research questions, for example changes in cell‐to‐cell contacts, higher cell seeding numbers, might be favored. However, if comparison to previously published CP data is required, choosing subconfluent conditions can be beneficial.

### Choosing Compound Concentrations, Compound Plate Layout, and Control Compounds

Screens can be performed either at single concentrations for primary hit detection, or using multiple concentrations per compound for hit confirmation and concentration‐response modeling. This choice often depends on the design of the compound library used and the number of compounds to be tested. Further, it may be beneficial to avoid overly cytotoxic concentrations of compounds to avoid having nonspecific cell toxicity effects compromising the specificity of the primary phenotypic readouts. For concentration‐response screenings in particular, we recommend running a range‐finding experiment prior to the main screen to identify cytotoxic compound concentrations using appropriate cytotoxicity/cell viability tests. This approach may be a bit more time consuming, but can significantly reduce costs associated with CPP. We have observed different levels of sensitivity with regard to cytotoxic concentrations between MCF‐7, HepG2, U2OS and RPTEC, with RPTEC being the least sensitive cell line (von Coburg et al., 2025a). Therefore, range‐finding experiments should be considered for each tested cell line individually. Use the lowest cytotoxic concentration from the range‐finding experiment as the highest starting concentration in the main CPP screen. This avoids nonspecific toxic effects and ensures sufficiently high exposure levels and, in particular, confidence in negative screening results.

If technically feasible, randomize all test, control, and reference compounds throughout the plate to minimize the impact of plate position effects. Compound layouts can be randomized within plates, with compounds and corresponding concentrations placed at varying well positions on the same plate across replicate runs of a screen. However, we recommend avoiding randomization across plates, i.e., subsets of compounds and corresponding concentrations being present on different plates for each replicate, as this complicates the data analysis routines.

When potential plate effects are sufficiently controlled, conducting biological replicate runs can be preferable to including technical replicates of test compounds per plate, due to the sufficiently high replicability between technical replicates, as previously demonstrated for the CPP method (von Coburg et al., 2025a). In our experience, running the CPP method in a single technical but four biological replicates usually results in robust and reliable phenotypic profiling data.

For reliable data analysis and interpretation, it is essential to reserve a sufficient number of wells on each screening plate to accommodate all relevant control conditions, including the solvent (vehicle) control, as well as a standard set of negative and positive control compounds. These include performance negative and positive controls (compound(s) needed for evaluating and monitoring CPP method performance and cell model responsiveness over time) and, if applicable, assay‐specific negative and positive controls (compound(s) needed for assay‐specific data analysis, such as standardization against reference profiles). Always include solvent control wells on each compound plate, because those are essential for per‐plate normalization (*z*‐score) of extracted raw feature values. We recommend using at least 24 solvent control wells per 384‐well compound plate to ensure statistically robust results. When using dimethyl sulfoxide (DMSO) as the solvent, we generally recommend in‐well concentrations of 0.1% (v/v); these may vary depending on the cell model and screening setup but should not exceed 0.5% (v/v) (as recommended by Cimini et al., 2023). Performance controls are used to monitor general CPP method quality and cell model responsiveness throughout the screen, independent of the research question or endpoint addressed in the screen. The performance negative control compounds should show no relevant effects on phenotypic profiles, whereas the corresponding performance positive control compounds should induce significant responses. As performance negative control compounds, we recommend sorbitol and saccharin, for which no or only subtle phenotypic response was observed in different cell lines in our previous data (von Coburg & Dunst, 2026; von Coburg et al., 2025a). As performance positive control compounds, we recommend tetrandrine, nocodazole, and/or cytochalasin D, which induce broad morphological responses affecting many different features (Odje et al., 2024; von Coburg & Dunst, 2026; von Coburg et al., 2025a). However, the selection of performance control compounds may vary between cell lines, depending on different susceptibilities and sensitivities. If possible, the corresponding compound concentrations of the performance controls should be kept constant between different cell lines and over time to enable direct comparison of cell responses.

Additionally, assay‐specific control compounds serve as a reference for an assay with clearly defined endpoints. Assay‐specific negative control (no relevant effects on phenotypic profiles) and positive control (significant effects on phenotypic profiles) compounds, and their concentrations, should be selected according to the specific readout that is addressed by the assay. For example, when specifically screening for tubulin‐targeting compounds, compounds that act as microtubule modulators should be included as a specific positive control (Akbarzadeh et al., 2022). However, exploratory screenings, where no defined endpoint is addressed, do not necessarily require assay‐specific negative or positive controls, whereas the aforementioned performance controls should always be included.

## Basic Protocol 1 SEEDING OF U2OS, MCF‐7, OR HepG‐2 CELLS IN MULTIWELL PLATES AND TREATMENT OF CELLS WITH COMPOUNDS

This protocol describes how to cultivate standard cancer cell lines such as U2OS, MCF‐7, or HepG‐2 and seed the cells in multiwell plates. It further describes how to stimulate the cells with test compounds.

### Materials

5 mg/ml collagen type 1, rat tail (Ibidi, cat. no. 50201)
Dulbecco's Balanced Salt Solution (DPBS), without Ca and Mg (PAN Biotech, cat. no. P04‐36500), sterile
Cell line, growing in a standard cell culture flask: U2OS (HTB‐96, ATCC); HepG2 (ACC 180, DSMZ), or MCF‐7/vBOS (Michigan Cancer Foundation‐7/variantBOS; Bischoff et al., 2020; Kornhuber, Dunst, Schönfelder, & Oelgeschläger, 2021)
Cell dissociation solution: e.g., Accumax Cell Dissociation Solution (PAN Biotech, cat. no. P10‐21250)
Cell growth medium appropriate for the cells used (U2OS, HepG2, or MCF‐7/vBOS; see recipes)
0.4% Trypan Blue stain (Invitrogen/Thermo Fisher Scientific, cat. no. T10282), for determination of cell viability
Assay‐ready compound plate containing test compounds and control compounds (see Support Protocol 1)

384‐well microplates for cell seeding, suitable for microscopy: e.g., PhenoPlates (Revvity, cat. no. 6057302)
Sterile cell culture materials: e.g., cell culture flask (TPP, cat. nos. TPP90076 [T75] and TPP90151 [T150])
Cell culture incubator, 37°C, 5% CO_2_, and high humidity (∼95% relative humidity; e.g., Memmert ICO50)
Serological pipets
Conical tube
Cell counting system: e.g., automated cell counter (Life Technologies/Thermo Fisher Scientific Countess II FL)
Multichannel pipet

Automated pipetting device suitable for 384‐well format: e.g., JANUS Automated Liquid Handling Workstation (Revvity Inc.)
Centrifuge (e.g., Thermo Fisher Scientific Megafuge 16R)

#### Coating of plates

For the HepG2 cell line, we recommend precoating 384‐well plates with collagen to ensure the growth of an even cell layer. For the U2OS and MCF‐7 cell lines, no collagen coating is required, so proceed directly with step 7.

1 Dilute collagen in sterile water to generate a 0.1 mg/ml solution.
  Check that collagen is dissolved completely in water and that no undissolved collagen clumps remain in the solution. To do so, cover an even surface (e.g., the surface of a cell culture flask or petri dish) with the 0.1 mg/ml collagen solution and check visually that the liquid layer appears smooth, without undissolved chunks of collagen.
2 Add 40 µl collagen solution to each well of the 384‐well microplate.
  Use a plate type with clear flat bottom that is compatible with your automated microscope of choice and suitable for cell growth.
3 Incubate plate 1 hr at room temperature.
4 Aspirate off collagen solution.
5 Wash plate once with sterile DPBS by adding 45 µl DPBS to each well of the plate.
6 Aspirate DPBS completely. Proceed to step 7 (cell seeding on the coated plates) on the same day.

#### Cell seeding

7 Using either HepG2, U2OS, or MCF‐7 cells growing in a standard cell culture flask, aspirate medium from cell culture flask and wash cell layer once with sterile DPBS.
8 Dilute Accumax cell dissociation solution 1:3 in DPBS and distribute over the cell monolayer.
9 Incubate at 37°C (in the incubator) for 5‐6 min, or until cells detach from surface.
10 Using fresh cell growth medium, wash the cells off the surface with a serological pipet.
11 Transfer cell suspension to a conical tube and count cell number using a cell counter.
12 Dilute cell suspension appropriately with cell growth medium to reach the desired cell concentration (Table 1).

**Table 1:** Suggested Seeding Conditions for Different Cell Lines

Cell line	MCF‐7/vBOS	U2OS	HepG2	RPTEC‐TERT1
Coating of assay plate	None	None	Collagen	Collagen
Growth period on plate prior to compound treatment	24 hr	24 hr	24 hr	10 days
Initial cell seeding number per well (values for orientation purpose only)	7,000‐14,000	3,000‐5,000	5,500‐7,000	6,000
Cell confluency after growth period	80‐100%	80%	80%	100%

13 Seed the initial cell number indicated in Table 1 into a well of a 384‐well plate (if working with HepG2 cells, a plate with collagen‐coated wells) using multichannel pipets or any other pipetting device of your choice. We usually seed 40 µl of cell suspension per well.
  Note that cell numbers suggested in Table 1 are for orientation purpose only and might need to be adjusted for individual experiment setups and compound stimulation durations. Generally, shorter compound stimulation times require more cells to reach the desired confluency.
14 Place assay plates in incubator (37°C, 5% CO_2_, ∼95% relative humidity) and allow cells to grow for 1 day.

#### Compound treatment of cells

15 Dissolve the compounds in the prepared assay‐ready compound plate (see Support Protocol 1) in cell culture medium. Add compounds to the cells using an automated pipetting device suitable for 384‐well format.
  Visually check that the compounds are completely dissolved in the cell culture medium before applying them to the cells. Make sure that no undissolved pellets of compounds remain at the well bottom of the assay‐ready compound plate. Adjust the mixing steps if needed. It is essential to transfer the precise volume of compounds to the cells in order to generate reproducible results. When using an automated pipetting device, make sure that the pipet tips emerge in the liquid of the compound plate and that no air is sucked into the tips during pipetting. Use tip‐touch at the well wall to ensure that the complete volume in the tips is transferred and no droplets remain stuck to the pipet tips.
16 Briefly centrifuge cell plate for 15 sec at 100 × *g*, room temperature (~22°C), to ensure that no droplets stick to the walls of the wells.
17 Place cells in an incubator at 37°C, 5% CO_2_, and ∼95% relative humidity. Incubate for 24‐48 hr, depending on the experimental setup chosen, before proceeding with the staining procedure (Basic Protocol 2).

## Support Protocol 1 PREPARATION OF ASSAY‐READY COMPOUND PLATES

After designing an appropriate plate layout that includes both the test compounds of interest, suitable control compounds, and solvent controls (see Strategic Planning), the next step is to prepare compound assay‐ready plates for use in the experiments, in which all compounds are spotted onto multiwell plates in advance of the experiment. Preparing all compounds at once for all replicates increases reproducibility between replicates and saves time on the day of the experiment. In this way, the compound assay‐ready plates can simply be stored at –20°C until the screening. To produce the assay‐ready plates, (acoustic) liquid handlers that precisely transfer very small volumes (nanoliter resolution) and enable randomized plate layouts for each replicate are preferred. On the day of the experiment, when the cells are to be treated with the test compounds, cell culture medium is added to the assay‐ready plates to dilute the compounds. From this compound solution, a defined volume is then transferred to the cells to expose them to the test compounds.

For example, 100 nl of a 1,000× compound stock solution (at 100% DMSO) are spotted on an assay‐ready plate. On the day of the experiment, 20 µl of medium is added to the assay‐ready plate, generating a 5× compound solution (at 0.5% [v/v] DMSO). Adding this 5× compound solution to the cells in a 1:5 dilution (10 µl of 5× compound solution added to cells grown in 40 µl of growth medium in the multiwell plate) results in the final 1 compound solution (at 0.1% [v/v] DMSO) to be tested on the cells. We recommend preparing at least one copy of the assay‐ready plate for each biological replicate of the screen and storing them at –20°C until use.

### Materials

Test compounds or compound library of interest for screening (as defined by the user)
Standard set of assay‐independent performance negative and positive controls: We recommend tetrandrine (Sigma‐Aldrich, cat. no. T2695), nocodazole (Sigma‐Aldrich, cat. no. M1404), and/or cytochalasin D (Sigma‐Aldrich, cat. no. C8273)
Solvent control: e.g., dimethyl sulfoxide (DMSO; ChemCruz, cat. no. sz‐358801)

384‐well V‐bottom PP microplates (Greiner, cat. no. 781280)
Liquid‐handling pipetting device: e.g., Echo acoustic liquid handler (Beckman Coulter).

1 Dissolve test and control compounds in an appropriate solvent (e.g., DMSO) at 1,000× the desired final test concentration, to minimize the final solvent background concentration to 0.1%.
2 Prepare assay‐ready plates by spotting 100 nl of each compound into a 384‐well V‐bottom plate using an appropriate liquid‐handling pipetting device.
3 Store assay‐ready compound plates at –20°C until use. Prepare for each replicate a single assay‐ready compound plate to avoid multiple freeze‐thaw cycles, which may affect compound stability.
4 Continue with Basic Protocol 1, step 15, adding 5× compound solution 1:5 to cells.

## SEEDING AND DIFFERENTIATING PRIMARY RPTEC‐TERT1 CELLS IN MULTIWELL PLATES

RPTEC‐TERT1 cells are TERT1‐immortalized renal proximal tubule epithelial cells and represent a primary kidney cell line (Wieser et al., 2008). To differentiate into a post‐mitotic multicell layer, these cells require a 10‐day growth time on the plate after seeding. During this period, the medium in the RPTEC plate should be replaced every 2‐3 days to provide a consistent nutrient supply. To ensure even growth of the cell layer, all cell culture flasks (T75 or T150) and multiwell plates should be coated with collagen (in a process analogous to steps 1‐6 of Basic Protocol 1) for regular cell culture.

### Additional Materials (also see Basic Protocol 1)

RPTEC‐TERT1 cells (Wieser et al., 2008) growing in collagen‐coated cell culture flasks
Fetal bovine serum (FBS; TICO Europe, cat. no. FBSEU500)
Cell growth medium for RPTEC cells (see recipe)
384‐well microplates for cell seeding, suitable for microscopy (e.g., Greiner Bio‐One, cat. no. 781866), precoated with collagen (see Basic Protocol 1, steps 1‐6)

#### Cell seeding

1 Aspirate medium from the cell culture flask and wash cell layer once with sterile DPBS.
2 Dilute Accumax 1:3 in DPBS and distribute over the cell monolayer.
3 Incubate at 37°C for 6 min or until cells detach from the surface.
4 Add 1 ml FBS to the cells to stop the Accumax reaction.
5 Using 10 ml of fresh cell growth medium, wash the cells off the surface with a serological pipet and transfer cell suspension to a conical tube.
6 Remove the additional FBS by spinning down the cell suspension for 5 min at 200 × *g*, room temperature (~22°C). Aspirate and discard the supernatant, and dissolve cell pellet in 10 ml fresh medium.
7 Count cell number using a cell counter.
8 Dilute cell suspension with cell growth medium to obtain a cell concentration of 150,000 cells/ml.
9 Seed 40 µl (containing 6,000 cells) of this cell suspension in each well of the 384‐well plate using a multichannel pipet.
  Note that the 384‐well plate should be coated with collagen in advance as described in steps 1‐6 of Basic Protocol 1.
10 Place cell plates in an incubator (37°C, 5% CO_2_, ∼95% relative humidity) and allow cells to grow for 10 days.
11 During this 10‐day growth time, change the cell growth medium of the RPTEC plate every 2‐3 days by gently aspirating the medium from the plate and adding fresh medium with a multichannel pipet.
12 After 10 days, proceed with compound treatment of the RPTEC cells as described in steps 15‐17 of Basic Protocol 1.

## Basic Protocol 2 STAINING AND IMAGING OF CELLS USING CELL PAINTING PLUS

This protocol describes the iterative staining‐elution cycle. In the first staining cycle, the cells are stained with the first set of dyes (including live‐cell dyes for mitochondria and lysosomes, as well as fixed‐cell dyes for RNA and actin), followed by imaging of cells using an automated fluorescence microscope. Subsequently, dyes are eluted using an elution buffer. In the second staining cycle, cells are re‐stained with a second set of dyes (including fixed‐cell dyes for DNA, Golgi, and ER) and re‐imaged. Generally, automated pipetting and plate‐handling devices to perform the staining protocols are recommended to avoid variation in the pipetting processes to the extent possible. However, for small‐scale or pilot experiments, manual pipetting can also be conducted.

### Materials

Cell assay plates (Basic Protocol 1 or Alternate Protocol)
2× live‐cell staining solution (see recipe)
2× fixation solution (see recipe)
Phosphate‐buffered saline (PBS; Santa Cruz, cat. no. sc‐362299)
2× cycle 1 staining and permeabilization solution (see recipe)
CPP elution buffer (see recipe)
2× cycle 2 staining solution (see recipe)

Automated liquid‐ and plate‐handling workstation: We use JANUS pipetting robot (Revvity Inc.), plate incubator (Liconic STX44 ICSA), microplate washer (BioTek 405LSUVS), and plate handler robot (Denso VS050A1‐AV6‐NNN)
Appropriate pipetting tips and liquid reservoirs for the respective automated pipetting device: We use sterile 384‐well, 30‐µL Janus MDT filter tips (Revvity, cat. no. 6001620) and 384 pyramid base geometry reservoirs for Janus G3 (Agilent, cat. no. 204612‐100)
High‐content imaging system equipped with 20× water objective and respective lasers and emission filter sets (Table 1): We use an Opera Phenix High Content Screening System (Revvity, HH14000000) equipped with Harmony software (v4.8), but other automated fluorescence microscopes equipped with same lasers and filter sets can also be used

#### Staining cycle 1

1 Gently aspirate the cell culture medium from the assay plate wells, leaving 15 µl of liquid in the wells, for example by using a microplate washer.
2 Pipet 15 µl of 2× live‐cell staining solution into each well (this will result in a total volume of 30 µl per well of 1× live‐cell dye solution). Generally, add staining solution to the middle of the well and enable tip‐touch on the wall of the well to ensure that no droplets remain stuck to the pipet tips.
3 Incubate plate for 30 min at 37°C protected from light.
4 Add 30 µl of 2× fixation solution to each well, for a final volume of 60 µl and concentration of 4% (v/v) PFA in the wells.
5 Incubate plate for 20 min at room temperature protected from light.
6 Wash assay plate three times with 100 µl PBS each time.
7 Aspirate the PBS from the assay plate wells, leaving 15 µl of liquid remaining in the wells.
8 Add 15 µl of 2× cycle 1 staining and permeabilization solution to each well.
9 Incubate for 30 min at room temperature protected from light.
10 Wash assay plate three times with 100 µl PBS. Leave ∼100 µl of PBS in the wells after the last wash‐step.
  If plates are not imaged immediately after staining, they can be stored at 4°C until imaging. However, we recommend proceeding with imaging within 2 days to minimize effects of variable dye stability over time and ensure reproducible results (von Coburg et al., 2025a).
11 Adjust the settings of the automated fluorescence microscope/high‐content imaging system: We recommend using a 20× water objective with 2 × 2 pixel binning.
  We compared results from 20× and 40× objective magnification and found high reproducibility between the two settings when comparable cell numbers are imaged (von Coburg et al., 2025a). We therefore decided to use 20× magnification, as it allows us to image more cells in a shorter imaging time compared to 40×.
12 Load a stained 384‐well plate into the microscope and adjust the height of the *z*‐planes so that the cell layer is in focus.
  Generally, imaging one optical section (z‐plane) should be sufficient. However, we recommend imaging two z‐planes with 2‐ to 4‐µm spacing, to make it possible to later choose the z‐plane that is most in focus for image analysis, or analyze the maximum‐intensity projection of both planes.
13 Choose the channels described in Table 2. Adjust the exposure times of the imaging channels as needed. The signal intensity inside the respective cellular compartment should be in the range of 4,000‐20,000 when using a 16‐bit camera (Table 2), and the signal‐to‐noise ratio (SNR) should be >5. Signal intensity must not reach saturation (e.g., avoid signal intensity beyond ∼65,000 when using a 16‐bit camera) to avoid loss of information and signal quantification failure. To prevent emission bleedthrough, which might severely impair profile specificity, we strongly recommend imaging all channels separately (channel sequence).
  We have used the Opera Phenix High Content Screening System (Revvity Inc.) in confocal mode for CPP image acquisition. However, CP has also been performed previously with microscopes in wide‐field mode (Bray et al., 2016), which might also be feasible for CPP, although this has not yet been tested.

**Table 2:** Details of Dyes and Imaging Channels Used in Cell Painting PLUS at an Opera Phenix Microscope*^a^*

Staining/imaging cycle	Dye	Channel name	Stained cellular structure	Optimal signal intensity range	Suggested exposure time*^b^*
1	Alexa Fluor Plus 405 Phalloidin	Hoechst 33342 (excitation: 405; emission: 435‐480)	Actin	6,000‐10,000	200 ms
	SYTO 14 Green Fluorescent Nucleic Acid Stain	Alexa 488 (excitation: 488, emission: 500‐550)	RNA	4,000‐10,000	200 ms
	MitoTracker Orange CMTMROS	Alexa 568 (excitation: 561; emission: 570‐630)	Mitochondria	10,000‐20,000	100 ms
	Cell Navigator Lysosome Staining Kit *NIR Fluorescence*	Alexa 647 (excitation: 640; emission: 650‐760)	Lysosomes	4,000‐10,000	100‐200 ms
2	Hoechst 33342	Hoechst 33342_2 (excitation: 405; emission: 435‐480)	DNA	4,000‐10,000	200‐400 ms
	Wheat Germ Agglutinin (WGA), Alexa Fluor 488 Conjugate	Alexa 488_2 (excitation: 488, emission: 500‐550)	Golgi	6,000‐15,000	40‐100 ms
	MitoTracker Orange CMTMROS	Alexa 568_2 (excitation: 561; emission: 570‐630)	Mitochondria	10,000‐20,000	100 ms
	Concanavalin A, Alexa Fluor 647 Conjugate	Alexa 647_2 (excitation: 640; emission: 650‐760)	Endoplasmic reticulum (ER)	6,000‐15,000	40‐100 ms

^*a*^ Table modified from von Coburg et al. (2025a), published under Creative Commons Attribution 4.0 International License (CC BY 4.0).

^*b*^ Exposure times represent values for orientation, but can be adapted to experimental variations in order to gain optimal signal intensities (with laser power = 100%).

14 Image two to six fields of view per well, depending on the confluency of the cells, to ensure the capture of at least 2,000‐2,500 cells in each solvent control well (Tromans‐Coia et al., 2023). As cell growth and confluency may differ between cell lines, adjust the number of fields of view per well to the respective cell lines.
  Imaging ∼2,500 cells/well for CPP was shown to ensure statistical robustness (von Coburg et al., 2025a).
15 After the imaging of staining cycle 1, check in the full‐plate view that the whole plate was stained and imaged correctly, as dyes from staining cycle 1 will be eluted in the following steps and cannot be re‐imaged.
  When using Opera Phenix with integrated Harmony software (Revvity Inc.), open the respective measurement in Harmony software, go to the “Image Analysis” tab, mark all wells of the plate in the plate layout on the right‐hand side of the user interface, right‐click, and choose “Overview – plate and well packed”. This will display a fast overview of the stained plate. If wells were not stained, they may appear dark in the overview image. Note that this step does not replace quality control, but rather provides a fast and easy way to get a first overview of the staining quality.

#### Elution

16 Wash plates three times with 100 µl/well of water each time. Afterward, aspirate the water from the assay plate wells, leaving 15 µl of liquid in each well.
17 Add 40 µl CPP elution buffer to each well.
18 Incubate plates for 10 min at room temperature.
19 Wash plates three times with 100 µl/well water each time.
20 Wash plates three times with 100 µl/well PBS each time. Leave ∼100 µl of PBS in the wells after the last wash step.
21 *Optional*: Imaging three to five random solvent control wells is recommended, so as to verify and document that the elution was successful. The remaining signal intensities should not exceed 1,500 when using a 16‐bit camera, and the SNR should be close to 1. The MitoTracker stain should not be eluted and should show a remaining signal intensity of ∼10,000 or beyond.

#### Staining cycle 2

22 Aspirate the PBS from the assay plate wells until 15 µl of liquid remains in each well.
23 Add 15 µl of 2× cycle 2 staining solution to each well.
24 Incubate for 30 min at room temperature protected from light.
25 Wash plates three times with 100 µl/well PBS each time. Leave ∼100 µl of PBS in the wells after the last wash step.
26 Image plates after staining cycle 2. Use exactly the same microscope settings as in the first imaging cycle but adjust the exposure times of the imaging channels to meet the recommended signal intensity ranges, as described in Table 2, and SNR >5.
  To make it possible to later combine the images from staining cycle 1 and 2, it is essential to use exactly the same imaging layout settings as in imaging cycle 1 in order to capture the exact same cells. The height and number of z‐planes, fields/well, channels, experimental layout, and plate type must be identical. Only the exposure time should be adapted to the different dyes used in staining cycle 2. All other parameter settings of the microscope should remain unchanged compared to staining cycle 1.
27 After successfully imaging all plates, continue with image analysis (Basic Protocol 3).

## Basic Protocol 3 IMAGE ANALYSIS

We previously compared the performance of image analysis pipelines built for the open‐source software CellProfiler (Carpenter et al., 2006) and the commercial software Harmony (Revvity Inc.) for analysis of CPP images (von Coburg et al., 2025a). Both pipelines extract hundreds to thousands of features (i.e., cellular readouts) from the images by measuring pixel intensity and distribution parameters. A feature is thereby a quantified phenotypic trait that typically comprises information about the analysis module (e.g., morphology, distribution, texture, intensity, and granularity) and the cellular region (e.g., nucleus, cytoplasm, membrane) for each imaging channel. For example, the feature *Intensity_Cytoplasm_Alexa 488 Mean* describes the mean intensity of the Alexa 488 imaging channel in the cytoplasm. However, not all CPP features are based on a single channel: for example, *Morphology_Cell Area* is measured from the overall size of the cell.

For Harmony, our image analysis pipeline delivers 894 preselected features, whereas the analogous CellProfiler pipeline provides 3,648 features (von Coburg et al., 2025a). We showed that both pipelines are generally applicable to CPP data (von Coburg et al., 2025a). However, using CellProfiler typically requires an additional time‐consuming step for image export from proprietary microscope software data formats. Furthermore, the numerous CellProfiler features also contain higher numbers of missing or constant values, thus needing more extensive filtering of features during the data cleaning procedure, which makes using CellProfiler somewhat less practical overall. In this protocol, we therefore describe the image analysis pipeline using Harmony. For more details on CPP image analysis using CellProfiler, we refer readers to von Coburg et al. (2025a) and Wedler, Körber, & Liu (2026).

In Harmony, an image analysis sequence is composed of a number of building blocks (BBs) and application‐specific building blocks (ABBs), each of them representing one step of the analysis pipeline. More details on the specific BB can be found in the Harmony manual (Revvity Inc.). Some key BBs of the Harmony CPP image analysis pipeline are listed in Table 3. The final outputs of the analysis from all BBs are collected for each single cell at the end of the analysis sequence and can be exported as a .txt file. The Harmony CPP image analysis pipeline presented here is described in detail by von Coburg and Dunst (2026). This may also serve as a guide for designing own customized pipelines. Note that using BBs in Harmony generally requires an additional license, which can be requested from Revvity Inc. Additionally, make sure to download and install the version of the ABB “*Add Channels 4i*” (see Materials) that matches the Harmony software version used.

**Table 3:** Key Building Blocks (BB) and Application Specific Building Blocks (ABB) Used in the Harmony CPP Image Analysis Pipeline*^a^*

Key step/building block	Description
*Input image*	Defines the original image as input area. Note that the Flatfield Correction parameter should be set to “Advanced”. The *Find Image Region* BB defines the whole image as output population.
ABB *Add Channels 4i*	Creates a multichannel image from all individual channels using the Mito channels from imaging cycles 1 and 2 as a *Channel for Matching* during the image registration process. Note that T1 and T0 of the measurement need to be set *as time window* (see Basic Protocol 3, step 8). Use the previously defined *whole image* as ROI population, and select and assign all other imaging channels with unique channel names (Table 2).
*Calculate Morphology Properties* and *Calculate Properties*	Optional step to quantify the image frame drift by calculating overlapping and non‐overlapping image area between channels from T0 and T1.
*Calculate Image*	Merges RNA and both Mito channels into a merged channel for the identification of cell outlines during the image segmentation process.
*Find Nuclei*	Identifies nuclei using the Hoechst channel.
*Find cytoplasm*	Identifies the cytoplasm and membrane region based on the RNA and Mito merged channels.
*Calculate position properties* and *select population*	Excludes cells touching the image border by calculating the distance between the cell and the image rim using the *calculate position properties* BB, and then selects cells with distance >0 using the *select population* BB.
*Calculate position properties*	Calculates the contact area of a cell with neighboring cells.
*Find spots*	Identifies the nucleoli inside the nucleus region based on the RNA channel.
*Select cell region*	Defines the RingRegion and MembraneRegion.
*Calculate properties*	Counts the number of nuclei per cell.
*Calculate Morphology/ Texture/ Intensity Properties*	Extracts the morphological (Standard, STAR), texture (SER, Haralick, Gabor), intensity, shape, and position features for all meaningful combinations of cell regions and channels.
*Define Results*	Calculates the median for each feature value obtained for each single cell (per‐object data), as well as the number of objects for all populations (e.g., cells and nucleoli).

^*a*^ Listed in the order in which they are applied in the pipeline.

### Materials

#### Software

Harmony image analysis software (Revvity Inc.) (we use Harmony v4.8, but applicability to higher Harmony software versions has also been confirmed);
ABB *Add Channel 4i* (Kirsch, 2025) for image registration in Harmony software versions v4.8, v4.9, v5.2, v5.3, as described in von Coburg & Dunst (2026) and von Coburg et al. (2025a) (we use *Add Channel 4i* ABB v1.0.4, available at https://doi.org/10.5281/zenodo.15119993)

##### Files

Harmony CPP image analysis pipeline integrating the customized *Add Channel 4i* application‐specific building block (ABB) for image registration as described in von Coburg & Dunst (2026) and von Coburg et al. (2025a) and available as Supplementary Data 19 in von Coburg et al. (2025b) at https://doi.org/10.5281/zenodo.14982928

##### Sample Files

CPP image data (Harmony measurements of CPP cycles 1 and 2)
*Optional*: CPP image data (available as Harmony measurement files) to serve as input for the Harmony CPP image analysis pipeline, if the user has not yet generated their own CPP images (these are data from a CPP screen using U2OS cells published in von Coburg et al., 2025a):
  Biological replicate 1 (imaging cycle 1), available at https://doi.org/10.5281/zenodo.17414605
  Biological replicate 1 (imaging cycle 2), available at https://doi.org/10.5281/zenodo.17491084
  Biological replicate 2 (imaging cycle 1), available at https://doi.org/10.5281/zenodo.17543163
  Biological replicate 2 (imaging cycle 2), available at https://doi.org/10.5281/zenodo.17550730
  Biological replicate 3 (imaging cycle 1), available at https://doi.org/10.5281/zenodo.17569912
  Biological replicate 3 (imaging cycle 2), available at https://doi.org/10.5281/zenodo.17579155
  Biological replicate 4 (imaging cycle 1), available at https://doi.org/10.5281/zenodo.17589464
  Biological replicate 4 (imaging cycle 2), available at https://doi.org/10.5281/zenodo.17596663

#### Combine images from two staining cycles into one multichannel image

1 Open the Harmony software. Go to “Settings” → “Data management” → “Combine Measurements”.
2 Click on the “+” symbol on the left lower corner of the dialog window to choose the measurements for one biological replicate plate from staining cycle 1 and staining cycle 2.
3 Set the measurement from staining cycle 1 as T0 and the measurement from staining cycle 2 as T1.
4 Write a plate name for the combined measurement. Press “start”. Note that this step will generate a new measurement file that is as storage‐intensive as the two single measurements together (e.g., a combination of two 40‐GB single measurements will result in an additional 80‐GB combined measurement file).
  Each plate needs to have a unique identifier. Labeling should follow a precise structure. We use, e.g., [first two letters of experiment owner's name]+[3‐digit experiment number]+[yy/mm/dd]+[two digit plate number], e.g., MW00923052901. Combined images could, e.g., be labeled with “Comb” (short for “combined”), followed by the number of the measurements that are combined, e.g., MW00923052901_Comb1_2. (Note that the plate name digits, in combination with the first underscore **_** in the plate name, will be used later in the Jupyter notebook to automatically detect plate IDs.)

#### Cell segmentation and feature extraction

5 Download the Harmony CPP image analysis pipeline (the Harmony‐Archive folder containing the analysis sequence is available as Supplementary Data 19 at https://doi.org/10.5281/zenodo.14982928). Then, open the Harmony software, choose “data management” → “read archive”, select the appropriate file path, and press start to import the pipeline (“EC019_230724_MCF7_CPP”) into your Harmony account.
6 Go to the image analysis tab in the Harmony software and load the combined measurement and the Harmony CPP image analysis pipeline. On the right‐hand side, choose one random well, field, and *z*‐plane from the plate layout panel.
7 If multiple *z*‐planes were imaged, select one or multiple *z*‐planes to be analyzed. Note that all *z*‐planes selected in this way will be analyzed separately. When the maximum‐intensity projection needs to be analyzed, proceed as described in step 10.
8 Select the two time points T0 and T1 and right‐click to choose “use as time window”.
9 Illumination correction is best performed using the advanced flatfield correction mode in Harmony. To do so, set the parameter “Flatfield Correction” to “Advanced” in the BB “Input Image” of the Image Analysis sequence.
10 Choose either “Individual Planes” or “Maximum Projection” as stacking process.
  Using automated confocal microscopes, samples can be efficiently imaged at multiple optical sections (z‐planes), which are subsequently analyzed either individually or as a maximum‐intensity projection. Previous analyses of CP data showed that phenotypic profile strength did not increase when using maximum‐intensity projections of multiple z‐planes for image analysis compared to using a single z‐plane in mono‐layered U2OS cells (Tromans‐Coia et al., 2023). Therefore, to save data storage and computational capacity, using a single z‐plane for image analysis is usually sufficient. However, for more complex cell lines growing in multilayers, using maximum‐intensity projections might help reduce problems with images being out of focus.
11 When using the image analysis pipeline for the first time or when applying it to a new cell line, visually check that the cell segmentation masks match your sample, especially for nucleus, cell region, and nucleolus segmentation, and adjust segmentation parameters if needed (see Table 3). If your microscope has different channel names from those listed in Table 2, the channel names need to be adjusted in the Harmony pipeline.
12 Go to the “evaluations” tab and select either single or batch mode. For single mode, select wells, fields, *z*‐plane(s), and, importantly, the two timepoints T0 and T1 to be included in the analysis. For batch mode, click the “+” symbol to load all combined measurements that should be analyzed. Note that in batch mode all wells, fields, and *z*‐planes of the combined measurements will be analyzed automatically, without any option to select subsets thereof. Start the evaluation by pressing the “start” button.
13 When the image evaluation is finished, export the evaluation results as a .txt file for each plate analyzed. To do so, go to “Settings” → “Data Management” → “Export data”. Choose as export method “Evaluation Results per Well and Object”. This will mean that the evaluation results will include the single‐cell data as well as well‐level aggregated results.
  The exported file will be named automatically as objects_population ‐ nucleoli.txt and objects_population ‐ cells.txt.
14 The exported evaluation result files can be analyzed further as described in Basic Protocol 4.

## Basic Protocol 4 DATA NORMALIZATION AND DATA QUALITY CONTROL

The customized *CPPAnalyzer* Jupyter notebook offers a flexible tool for both CP and CPP data analysis, including plate‐wise data normalization and optional data cleaning steps. Jupyter (Kluyver et al., 2016) is an open‐source, web‐based computing platform with which different computing languages can be used (e.g., R). Here, R code is used to calculate robust *z*‐scores using the median and median absolute deviation (MAD) of each feature for each well. In this way, the responses of cells (feature values) exposed to test chemicals are scaled against the solvent control cells using established normalization methods, as summarized in Table 4. As batch and plate position effects have previously been observed in CP‐based data (Arevalo et al., 2024; Caicedo et al., 2017), the *CPPAnalyzer* Jupyter notebook integrates several QC steps to detect them. Several parameters in the notebook can be adjusted by the user, offering a flexible tool that can be adapted to individual use cases. Further, a compound layout file is used to assign to each well the respective treatment condition. In case wells need to be excluded from analysis, for example due to known pipetting errors or other artifacts, this can be done by listing those wells in the respective “wells to exclude” table.

**Table 4:** Normalization Methods Available in the *CPPAnalyzer* Jupyter Notebook

Normalization method	Explanation	Reference	Setting in the Jupyter notebook
All‐cell normalization	First, feature values of each single cell are normalized to the median and MAD of pooled solvent control cells from the plate. Second, single‐cell *z*‐scores are aggregated to well levels.	Nyffeler et al. (2020); von Coburg et al. (2025a)	norm = “CW”
Well‐level normalization	First, single‐cell feature values are aggregated to well level using median or mean. Second, well‐level data are normalized to the median and MAD of solvent control wells from the plate.	Bray et al. (2016); Cimini et al. (2023)	norm =“W”
*B*‐score normalization	First, single‐cell features are aggregated to well level using the median or mean. Second, the *loess* function from the R package *loess* is applied to remove the effect of the variables “Row” and “Column” from the aggregated data as suggested by Caicedo et al. (2017). Last, the corrected data are normalized to the median and MAD of solvent control wells from the plate.	Caicedo et al. (2017); Mpindi et al. (2015)	norm = “Bnorm”

### Materials

#### Hardware

Workstation or high‐performance computer (HPC) system (with at least 25 GB RAM for analysis of CPP data using the *CPPAnalyzer* Jupyter notebook)

##### Software

Jupyter notebook software (Kluyver et al., 2016), https://www.jupyter.org/
R (R Core Team, 2024), https://www.R‐project.org/ (we use R v.4.2.0)
R packages RColorBrewer (Neuwirth, 2022), tools (R Core Team, 2024), data.table (Barrett et al., 2025), foreach (Microsoft Corporation & Weston, 2022), grid (R Core Team, 2024), gridExtra (Auguie, 2017), ggplot2 (Wickham, 2016), doParallel (Microsoft Corporation & Weston, 2022), matrixStats (Bengtsson, 2025), pheatmap (Kolde, 2025), and readxl (Wickham & Bryan, 2025)

##### Files

*CPPAnalyzer* Jupyter notebook (CPPAnalyzer.ipynb file) and corresponding R source code (https://doi.org/10.5281/zenodo.18385218)
Compound layout file (see step 2 of this protocol; an example file can be found at https://zenodo.org/records/18385218 )
Table to exclude wells with known experimental error from analysis (see step 3 of this protocol; an example file can be found at https://doi.org/10.5281/zenodo.18385218)

##### Sample Files

Harmony image analysis result output files (.txt format; see step 13 of Basic Protocol 3)
*Optional*: Harmony image analysis result output files (https://doi.org/10.5281/zenodo.18184315) to serve as input for the *CPPAnalyzer* Jupyter notebook, if the user has not yet generated their own CPP data

1 Set up the folder structure as illustrated in Figure 1C, and save the Harmony image analysis results output files (.txt format) on the high‐performance computer system or workstation. As the Jupyter notebook will access the chosen file path to read the respective files automatically, we recommend keeping the file structure as described in Fig. 1C.
  Example folder structure: [Screen ID]/[plate name]/evaluation 1/objects_population ‐ nucleoli.txt and objects_population ‐ cells.txt
  Prepare the following three Excel files (step 2‐4) and save them under the main [Screen ID] folder. The CPPAnalyzer Jupyter notebook will automatically access these files to retrieve this information.
2 Prepare a “compound layout” Excel file that lists, for each plate and each well position, the respective compound and concentration. In the column‐based layout file, all wells of all plates are listed in one table. The layout file should contain at least the following information: “Column”, “Row”, Compound′, “Concentration”, “Compound_ID”, “Plate”, “PlateNumber”, “ScreenPlateID”, “Replicate”, and “Trep” (technical replicate; see file Compound_layout_columnbased.xlsx at https://doi.org/10.5281/zenodo.18385218 as an example).
3 Prepare a “wells to exclude” Excel file that lists, for each plate, all wells with known experimental errors that should be excluded from the analysis (for example, due to known pipetting errors during the staining procedure). The column headers of the Excel sheet should represent the plate names. Under each plate name/column, the wells to be excluded from the respective plate should be listed. Note that all plate names need to be listed here, even when no well is to be excluded from the respective plate (just list the plate name as column header, without listing any wells underneath). This also makes it possible to limit the analysis to a subset of the wells per plate, if deemed useful. An example file, Table_to_exclude_wells.xlsx, can be found at https://doi.org/10.5281/zenodo.18385218.
4 Prepare a “planes” Excel file that states, for each plate, the optical section (*z*‐plane) that should be analyzed. This is useful when several z‐planes have been imaged and analyzed in Harmony (e.g., in batch mode) but only one is meant to be used for profile generation. If one or more planes have been analyzed in Harmony, the Harmony image analysis result output files will contain a column called “plane.” The respective Excel file should indicate, for each plate, which *z*‐plane is to be used for analysis in the Jupyter notebook. See the file Table_planes.xlsx at https://doi.org/10.5281/zenodo.18385218 as an example.
  The file Table_planes.xlsx must be prepared in any case. However, if image analysis was done using maximum‐intensity projection of several planes, there will be no “plane” column in the Harmony image analysis results output files. In this case, the file Table_planes.xlsx will be ignored by the CPPAnalyzer Jupyter notebook.
5 Create a new, separate folder where the result files will be saved later. Save the CPPAnalyzer.ipynb file in this results folder with your preferred name. In this way, the notebook is saved along with the results in the same folder and allows the user later to retrace chosen parameter settings in the notebook if needed.
6 Open the *CPPAnalyzer* Jupyter notebook within the aforementioned folder. Instructions on how to open and run the Jupyter notebook depending on the operation system can be found on the Jupyter website (https://docs.jupyter.org/en/latest/running.html).
7 Indicate the locations of the input data folder and the output folder for results in the first field of the notebook, as well as the location of the corresponding R source code.
8 Change the parameter variables (Table 5) to customize your analysis, if needed, and save the notebook. The variable “norm” will define which normalization method is used (Table 4).

**Table 5:** Parameters Adjustable by the User in the *CPPAnalyzer* Jupyter Notebook User Interface

Variable name	type	Explanation
screenid	String	All output files will have the screenID tag included in the file name.
minarea	numerical	If a filter for the size of nuclei is to be used, this parameter defines the lower limit of the nuclei size; all cells with nuclei smaller than this value will be excluded from analysis. If no filter is desired by the user, set the parameter to zero.
maxarea	numerical	This parameter defines the upper limit of the nuclear size filter; all cells with nuclear area greater than this threshold will be excluded from analysis. If no filter is to be used, set the parameter to infinity (“Inf”).
controlid	String	Defines the name of the solvent control used for *z*‐score calculation (e.g., DMSO). This must match the name listed for the solvent control in the compound layout file.
propNAfea	Numerical (value between 0 and 1)	Proportion of missing values tolerated in each column (each column corresponds to one morphological feature). If a column has more missing values than the proportion stated here, the column (feature) will be maintained but set to “NA”.
propNAcell	Numerical (value between 0 and 1)	Proportion of missing values tolerated in each row (each row corresponding to a single object). If a row has more missing values than the proportion stated here, the row will be excluded from analysis.
robustNorm	Logical (TRUE/FALSE)	If this parameter is set to “TRUE”, the median/MAD is used for *z*‐score calculation. With the parameter set to “FALSE”, mean/SD will be used instead. Note that features *number of nucleoli per cell* and *number of cells per well (Cell_TotalNumbers)* (which are features with discrete values) will always be normalized using mean/SD independently of the parameter *robustNorm*.
Nucleoli	Logical	If this parameter is set to “TRUE”, the analysis will include nucleoli‐related features (that are in the separate file *objects_population – nuceloli.txt)*
ncharLayoutno	numerical	Denotes the number of characters at the end of (or before the character “_” in) the *platefolder* parameter that defines the plate number: e.g., if the *platefolder* parameter is MW00924052901 and *ncharLayoutno* = 2, the last two characters (“01”) will be used as the plate number.
featag	string	Tag that is included in all morphological feature names. This is usually defined by the “Output population” in the Harmony evaluation pipeline: e.g., if the output population was defined as “Cells” in the Harmony evaluation, each feature will have the prefix “Cells”. If *featag* = “Cells”, all columns with the tag “Cells” in the column header will be used for data analysis.
featagNuc	string	Same as *featag* but for the Nucleoli file (if Nucleoli = TRUE).

**Figure 2:**
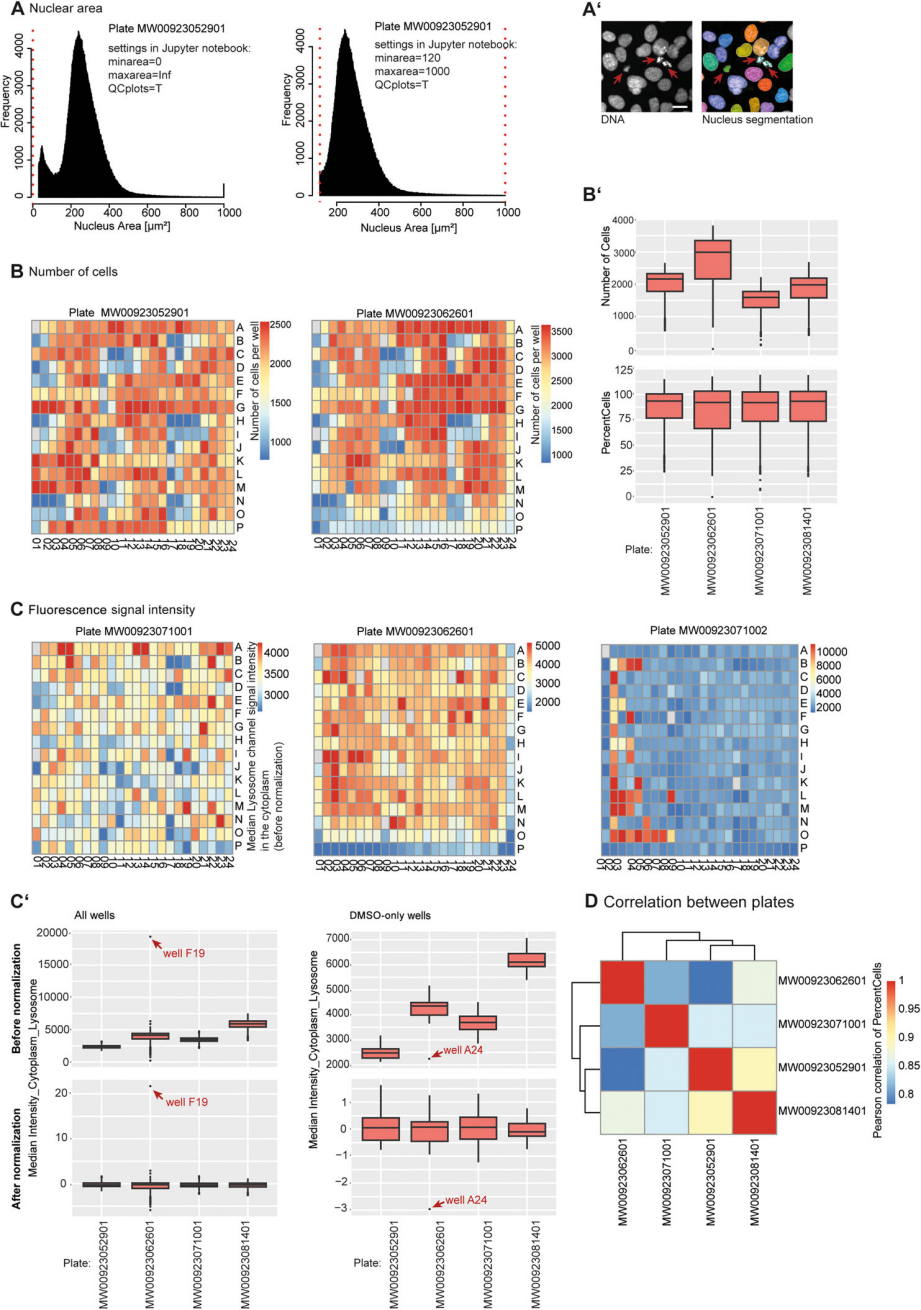
The *CPPAnalyzer* Jupyter notebook generates different diagnostic plots for data QC purposes. (**A**) The nuclear area filter can be applied to remove small fragments or mis‐segmented cells that, for example, may be observed in U2OS cells upon compound treatment. Histograms show the distribution of single cells for the nuclear area (µm^2^) feature. Data include all wells of the respective plate. Values >1,000 are set to 1,000. Red dotted lines indicate exemplary thresholds set for the nuclear size. Either no filter is applied (left) or objects with nucleus <120 µm^2^ (minarea) and >1,000 µm^2^ (maxarea) are discarded from analysis for the same plate (right). The parameter “QCplot” indicates whether or not the diagnostic plots for nuclear size are generated by the *CPPAnalyzer* Jupyter notebook. (**A′**) DNA staining in U2OS cells and segmentation of nuclei (using Harmony software) in DMSO solvent control wells. Arrows point to small fragments (presumably apoptotic cells or nuclear fragments) with nuclear area <120 µm^2^; these can be excluded from analysis using the nuclear size filter. Scale bar, 20 µm. (Images are not part of the *CPPAnalyzer* Jupyter notebook output files.) (**B**) Investigating plate position effects and number of cells per well. Heatmaps show the number of cells per well for a plate with non‐randomized compound layout, revealing some wells with low cell count (blue color) due to compound treatment. (**B′**) Number of cells and percentage of cells (relative to solvent control wells) for different plates. This makes it possible to spot differences between plates or replicates. For example, plate MW00923062601 has more cells per well and a higher intraplate variability than other plates. (**C**) Heatmaps show the signal intensity of the lysosome channel per well in different plates before normalization. The left plate shows no pronounced plate effects, whereas the middle plate shows edge effects, with lower signal intensity in row P and columns 1 and 24. The right plate exemplifies artifacts due to pipetting error during the application of staining solution, e.g., due to blocked pipet tips. (**C′**) Signal intensity of the lysosome channel in different biological replicates of a plate before and after normalization for all wells (left) and solvent control (DMSO) wells only (right). This enables the identification of differences between plates and outlier wells. For example, well F19 shows higher lysosome channel signal than other wells, whereas well A24 shows lower intensity than other DMSO wells. Plots in C and C′ are generated for all channels by the *CPPAnalyzer* Jupyter notebook, but only the lysosome channel is shown here, as an example. (**D**) Correlation matrix showing the Pearson correlation score of the feature *PercentCells* (relative to solvent control) between different plates. This plot can detect differences between different batches of screening plates. For example, plate MW00923062601 is slightly less correlated with other plates than the rest. Data used to generate the plots in this figure were previously published in von Coburg et al. (2025a).

9 To start the analysis, choose “Cell” → “Run all” in the menu.
10 When the notebook has finished its analysis, it will automatically save the unnormalized data and the normalized data (aggregated to median or mean per well) in the indicated results folder.
11 Examine the QC plots that are generated for possible batch or plate position effects (Fig. 2). See Supporting Information Table 1 for an overview of the plots and files that are automatically generated by the *CPPAnalyzer* Jupyter notebook. Also see the Understanding Results section for more information.

12 If needed, adjust parameter settings, for example nuclear size filter or wells to be excluded, and run the *CPPAnalyzer* Jupyter notebook again.
13 The normalized data can then be fed into the *CPPManager* KNIME workflow for further analysis and data visualization (Basic Protocol 5).

## Basic Protocol 5 VISUALIZATION OF DATA USING *CPPManager* KNIME WORKFLOW

**Figure 3:**
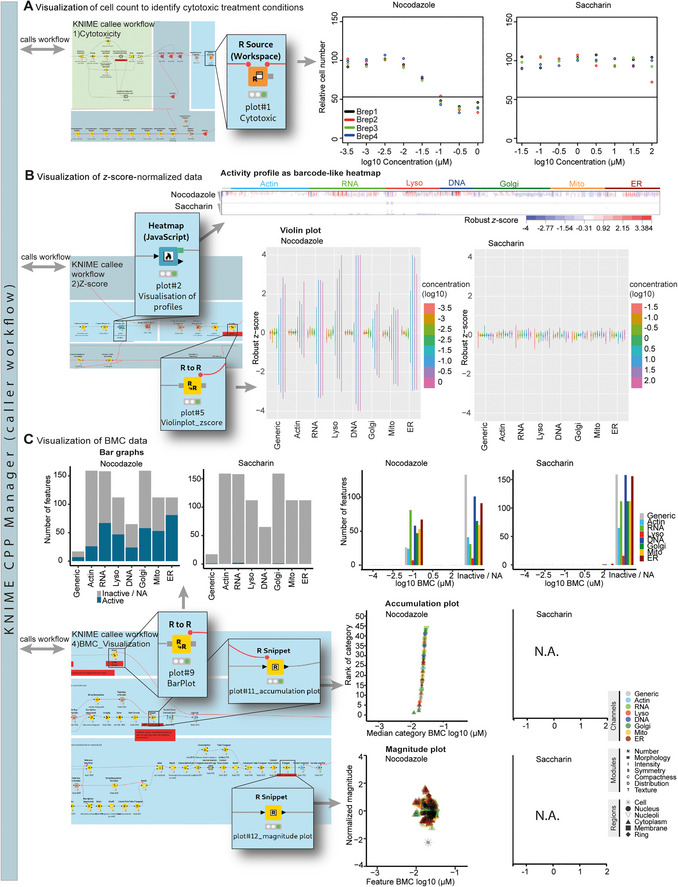
The automated *CPPManager* KNIME workflow (caller workflow) calls different subordinate KNIME workflows (callee workflows), which employ snippets of R code to process and visualize CPP data. The plots show results from a CPP screen using U2OS cells (the original data set was published in von Coburg et al., 2025a) with *n* = 4 biological replicates, each comprising *n* = 3 technical replicates. Data were processed using the *CPPManager* KNIME workflow. The plots show nocodazole as an example of a performance‐positive control and saccharin as an example of a performance‐negative control compound. (**A**) The first KNIME workflow, “Cytotoxicity,” shown at left, identifies cytotoxic treatment conditions based on the cell count relative to the solvent controls. It includes an R Source node (shown as insert) that plots the cell number relative to solvent control (in % cell count) for different concentrations tested (in µM). Different colors represent biological replicates (Brep), each of which is the median from three technical replicates. The horizontal black line indicates the cytotoxicity threshold (e.g., 50% cell count), which was defined by the user as an interactive variable of the *CPPManager* KNIME workflow. (**B**) The second *CPPManager* KNIME workflow, “z‐score visualization,” includes different types of plots to visualize the normalized data and generate phenotypic profiles. The heatmap node plots all features for each compound as robust *z*‐scores in a barcode‐like heatmap (median of 4 biological and 3 technical replicates). Features are ordered by imaging channel. Triangles indicate decreasing test concentrations. The “R to R” node includes an R script that generates violin plots to show the distribution of robust *z*‐scores of all features per imaging channel. Colors represent the different tested concentrations. (**C**) If concentration‐response screening data have been generated, benchmark concentration (BMC) modeling can be performed. The workflow “BMC_Visualization” shows the BMC modeling results in several different plot types. The bar graph shows the number of active and inactive features for different channels as well as for the different concentrations. The accumulation plot shows feature categories in which at least 30% of features show a response. Feature categories are ranked according to the median BMC of all single features included in this category. The magnitude plot shows the maximum effect size (magnitude) for each single feature, which is the maximum robust *z*‐score. Note that for saccharin, no plots can be shown because there are no active feature categories with >30% active features.

KNIME is an open‐source and free‐to‐use software tool and can be downloaded from the KNIME website https://www.knime.com/downloads. The *CPPManager* KNIME workflow combines different data processing and visualization steps by controlling and connecting several individual KNIME workflows. The superordinate Manager workflow (“caller workflow”) calls (activates) the subordinate “callee” workflows. Each callee workflow performs a designated analysis task, such as filtering of cytotoxic treatment conditions or visualization of normalized *z*‐score data (Fig. 3). If concentration‐response data have been generated during the CPP screen (multiple concentrations per compound tested), BMCs can be calculated for each feature based on the integrated *tcplfit2* R package (Sheffield, Brown, Davidson, Friedman, & Judson, 2022). Each feature for which a BMC was calculated will be subsequently assigned to a predefined CPP feature category. Each of the 64 CPP feature categories represents a combination of channel, cellular compartment, and analysis module (e.g., *Actin‐Cytoplasm‐Texture*), as described previously (von Coburg et al., 2025a; Supporting Information Table 3). The BMCs of features and feature categories are then visualized using accumulation and magnitude plots to support qualitative data interpretation (Fig. 3C).

As the *CPPManager* KNIME workflow runs snippets of R code, it is necessary to have R, including the required libraries, installed on your machine.

### Materials

#### Software

KNIME Analytics Platform (Berthold et al., 2007), https://www.knime.com (we use KNIME version 5.4.3); a user guide to KNIME installation and operation can be found at https://docs.knime.com/ap/5.4/, and earlier KNIME versions can be downloaded from https://www.knime.com/downloads/previous
KNIME extensions: KNIME Interactive R Statistics Integration (https://hub.knime.com/knime/extensions/org.knime.features.r/latest), KNIME Parallel Chunk Loop Nodes (https://hub.knime.com/knime/extensions/org.knime.features.virtual/latest), and KNIME HCS Tools (Max Planck Institute of Molecular Cell Biology and Genetics, 2014) which can be found at https://hub.knime.com/mpicbg-tds/extensions/de.mpicbg.tds.knime.hcstools.feature/latest
R (R Core Team, 2024), https://www.R‐project.org/ (we use R version 4.2.0)
R packages: data.table (Barrett et al., 2025), doParallel (Microsoft Corporation & Weston, 2022), dplyr (Wickham, François, Henry, Müller, & Vaughan, 2023), foreach (Microsoft Corporation & Weston, 2022), ggpubr (Kassambara, 2025), gg.gap (Lou, Zhang, Lvy, & Jin, 2019), ggplot2 (Wickham, 2016), stringr (Wickham, 2023), matrixStats (Bengtsson, 2025), pheatmap (Kolde, 2025), RColorBrewer (Neuwirth, 2022), Readxl (Wickham & Bryan, 2025), Rserve (Urbanek, 2025), Scales (Wickham, Pedersen, & Seidel, 2025), Svglite (Wickham, Henry, et al., 2025), tcplfit2 (Sheffield et al., 2022), tibble (Müller & Wickham, 2023), and tidyverse (Wickham et al., 2019)

##### Files

*CPPManager* KNIME workflow (for KNIME v5.4) as described below, available as a .knar file at https://doi.org/10.5281/zenodo.18184356
An Excel file grouping the compounds or treatment conditions into different subgroups for analysis, if needed (see step 2 of this protocol; for an example, see the file Example_Compound_Group.xlsx, available at https://doi.org/10.5281/zenodo.18184356)

##### Sample Files

Normalized CPP data (well level) as .csv file
*Optional*: Normalized CPP data (https://doi.org/10.5281/zenodo.18184315) to serve as exemplary input for the *CPPManager* KNIME workflow, if the user has not yet generated their own CPP data

1 Download the *CPPManager* KNIME workflow, included in the CPP_Manager_KNIME_workflow.knar file, from https://doi.org/10.5281/zenodo.18184356. Open KNIME, go to the file menu, choose “Import KNIME workflow”, and select the appropriate .knar file. Note that all KNIME callee workflows and the “Manager” workflow (caller workflow) must be located in the same subfolder within your local KNIME workspace.
  Note that you may need to adjust settings of the R nodes (e.g., R snippet, R source, and R to R nodes) in KNIME depending on your R environment. Open the respective node configuration window, go to advanced and choose “Specify path to R home” to set a path to the root folder of the R installation. Alternatively, use the default settings by deselecting “Overwrite default path to R home”. If you wish to use a Conda environment to manage R, including all required R packages, connect the respective R nodes with a Conda Environment Propagation node, which is part of “KNIME Conda Integration” feature provided by KNIME AG, Zurich, Switzerland (https://hub.knime.com/knime/extensions/org.knime.features.conda/latest).
2 Create an Excel file that groups the compounds or treatment conditions into different subgroups for analysis (i.e., Example_Compound_Group.xlsx). This can be useful when several compounds or compound libraries belonging to different projects have been tested side by side in the same screen, but separate plots and data files need to be generated for individual data analysis. In such a case, the *CPPManager* KNIME workflow will save the results for each compound group in a separate subfolder. If you do not wish to separate your compounds into subgroups, just list all compounds in one group file. See the file Example_Compound_Group.xlsx available at https://doi.org/10.5281/zenodo.18184356 as an example. To avoid problems arising from special characters in chemical naming, using a compound ID instead of the compound name is strongly recommended. Such compound IDs should preferentially be used as unique identifiers instead of parsing compound names through the KNIME pipeline.
3 In KNIME, open the “Manager” workflow in the folder “CPP_Harmony_Automated KNIME manager workflow”.
4 Read the compound group file using the “Excel reader” node and the normalized data using the “CSV reader” node as indicated in the workflow annotations.
5 Execute and open the user interface component by right‐clicking on the node and choosing “Execute and Open Views”. This will open an interactive user interface where several parameters can be adjusted.
6 In the user interface, enter the screen ID.
  The screen ID is a unique identifier for your CPP screen experiment. It will, for example, be documented automatically in the analysis details sheet (see Supporting Information Table 2) to help you trace which data were analyzed with the workflow. We usually use screen ID labels composed of two letters, for the initials of the experiment owner, and a three‐digit experiment number: e.g., MW009.
7 In the user interface, enter the file path to a folder where the result files will be saved later.
8 Enter a numerical value for the cytotoxicity threshold in the user interface. This value defines the minimum acceptable cell count (expressed as a percentage relative to the solvent control). If the percentage in at least half of the replicate screens falls below the defined threshold, the condition will be classified as cytotoxic and excluded from downstream analysis. Cell counts are determined based on the number of objects (i.e., nuclei) identified in the Harmony analysis sequence.
  The definition of the cytotoxicity threshold strongly depends on the experimental setup, cell line, cell population doubling time, and compound exposure time, and it can be adjusted as needed. For example, in earlier CP‐ or CPP‐based studies using U2OS or MCF‐7 cells, treatment conditions resulting in <50% of cell count compared to the solvent control were regarded as cytotoxic and excluded from analysis (Nyffeler et al., 2023; von Coburg et al., 2025a).
9 Choose the analysis based on data type: For concentration‐response data sets (when multiple concentrations per compound have been tested), additional BMC modeling can be performed. For single‐dose screening data, the BMC modeling step should be omitted by selecting the corresponding option in the user interface.
10 By clicking the respective option in the user interface, choose whether you would like to apply filtering options for BMC modeling, as described previously (Feshuk et al., 2023; Nyffeler et al., 2023; US EPA, 2012). In that case, the filtering will define BMCs as inactive and replace the calculated value with a missing value when the following conditions apply: a hitcall of ≤0.9, missing BMDU/BMDL values, or a BMC value greater than the maximal nontoxic concentration (BMC_high_).
  Hitcall is a parameter generated by the tcplfit2 R package, indicating the probability of a treatment condition being active. A hitcall ≤0.9 may indicate noisy or borderline response with overfit curves and less reproducibility, and thus hitcall >0.9 is used as one filter in CPPManager for active BMC (Feshuk et al., 2023). BMDU (upper bound) and BMDL (lower bound) indicate the 95% confidence interval of the BMC. If BMDU and BMDL values are missing, this implies insufficient curve fitting, or missing data points, or inactivity of the compound.
11 Additionally, define in the user interface, if you would like to apply the following modification to calculated BMC values lower than the lowest tested concentration (BMC_low_). In that case, BMC_low_ values are set to a generic value that is a half‐log concentration smaller than the lowest tested concentration (see Statistical Analysis) to visually separate features in the data plots for which no reliable BMCs could be calculated by interpolation, as described previously (Nyffeler et al., 2021; von Coburg et al., 2025a; also see Statistical Analysis).
12 The parameter “Chunk for Plots” defines how many chunks (blocks) will be processed in parallel. This does not affect the analysis results, but choosing a larger number of parallel chunks will speed up the computation. This increases CPU usage by distributing workflow computations across multiple CPUs. In our experience, selecting 5 chunks give a reasonably fast data processing without generating an excessive workload to the server.
13 The parameter “plot height” defines the dimensions in inches for the saved output graphs (applicable for the output graphs #3 and #4 as listed in Supporting Information Table 2).
14 Click “Apply temporarily” and close the window.
15 Execute all executable nodes by clicking on the double green arrow in the top menu bar to run all downstream nodes and execute the workflow. When the workflow is finished, all results will be automatically saved, including .txt files along with all raw data (normalized *z*‐score data and BMC modeling results). Generated plots will be saved as .svg and .pdf files.
16 Upon finishing the *CPPManager* workflow successfully, the user will receive an automated notification via e‐mail. This can be useful when analyzing large amounts of data that take longer to process. To indicate the e‐mail address to which this email should be sent, enter the address in the *Table Creator* node at the end of the Manager workflow, as indicated in the respective node annotation in the workflow.

## REAGENTS AND SOLUTIONS

### Cell growth medium for HepG2 cells

465 ml DMEM (1.0 g/L glucose, sodium pyruvate, no glutamine, no phenol red; 93% [v/v] final, Gibco/Thermo Fisher Scientific, cat. no. 11880‐028)
25 ml fetal bovine serum (FBS; 5% [v/v] final, Sigma, cat. no. BCBQ7890V)
5 ml penicillin‐streptomycin mix (1% (v/v) final = 100 µg/ml streptomycin, 100 U/ml penicillin final, PanBiotech, Cat. no. P06‐07100)
5 ml GlutaMAX (1% (v/v) final = 2 mM final, Gibco/Thermo Fisher Scientific, Cat. no. 35050‐061)
Mix well.
Store up to 1 month at 4°C.

### Cell growth medium for MCF‐7/vBOS cells

465 ml DMEM (1.0 g/L glucose, sodium pyruvate, no glutamine, no phenol red; 93% [v/v] final, Gibco/Thermo Fisher Scientific, cat. no. 11880‐028)
25 ml fetal bovine serum (FBS; 5% [v/v] final, Biochrom/Merck, cat. no. S0615; estradiol levels: 22.3 pg/ml)
5 ml penicillin‐streptomycin mix (1% [v/v] final = 100 µg/ml streptomycin/100 U/ml penicillin final, PanBiotech, cat. no. P06‐07100)
5 ml GlutaMAX (1% (v/v) final = 2 mM final, Gibco/Thermo Fisher Scientific, Cat. no. 35050‐061)
Mix well.
Store up to 1 month at 4°C.

### Cell growth medium for RPTEC

483 ml DMEM, no glucose, sodium pyruvate, or glutamine (48.3% [v/v] final; Gibco/Thermo Fisher Scientific, cat. no. 11966025)
483 ml Ham's F‐12 Nutrient Mix (48.3% [v/v] final; Gibco/Thermo Fisher Scientific, cat. no. 21765029)
5 ml penicillin‐streptomycin mix (50 µg/ml streptomycin and 50 U/ml penicillin final, PanBiotech, cat. no. P06‐07100)
10 ml of 200 mM GlutaMAX stock (2 mM final; Gibco/Thermo Fisher Scientific, Cat. no. 35050‐061)
10 ml insulin‐transferrin‐sodium selenite mix (∼5 µg/ml insulin, ∼5 µg/ml transferrin, and ∼5 ng/ml sodium selenite final; Sigma‐Aldrich/Merck, cat. no. I1884‐1VL)
5 ml FBS (0.5% [v/v] final; TICO Europe, cat. no. FBSEU500)
1 ml of 10 µg/ml hEGF stock (10 ng/ml final; Sigma‐Aldrich/Merck, cat. no. E9644‐2MG)
1 ml hydrocortisone (36 ng/ml final; Sigma‐Aldrich/Merck, cat. no. H0135‐1MG)
2 ml of 50 mg/ml G418 stock (0.1 mg/ml final; Roche, cat. no. 04727894001)
Mix well.
Store up to 1 month at 4°C.

### Cell growth medium for U2OS cells

470 ml DMEM (4.5 g/L glucose, sodium pyruvate, glutamine, no phenol red, 94% [v/v] final; PanBiotech, cat. no. P04‐03588)
25 ml fetal bovine serum (FBS; 5% [v/v] final, Sigma, cat. no. BCBQ7890V)
5 ml penicillin‐streptomycin mix (1% (v/v) final = 100 µg/ml streptomycin, 100 U/ml penicillin final; PanBiotech, cat. no. P06‐07100)
Mix well.
Store up to 1 month at 4°C

### SDS stock solution, 10% (w/v)

Weight 5 g sodium dodecyl sulfate (SDS), ultra‐pure (Carl Roth, cat. no. 2326.4), into a suitable cylinder or bottle. Add deionized water and mix to obtain a 50 ml final volume.

Use the 10% (w/v) SDS stock solution to prepare the CPP elution buffer.

### CPP elution buffer

The elution buffer consists of 0.5 M l‐glycine and 1% SDS in water with pH adjusted to 2.5.

To prepare 100 ml elution buffer, dissolve 3.754 g l‐glycine (Sigma‐Aldrich, cat. no. G8898‐1KG) in 50 ml deionized water. Add 10 ml of 10% (w/v) SDS stock solution (see recipe) to 1% (v/v) SDS final. Adjust the pH to 2.5 using hydrochloric acid (HCl) and a pH meter. Fill to 100 ml final volume with water.

Sterile filter the solution using a 0.22‐µm‐pore‐size filter to avoid crystals, e.g., using a bottle‐top vacuum filter system with a 0.22‐µm filter (Sigma‐Aldrich, cat. no. 430769).

Store up to 1 month at room temperature (20‐22°C).

### Fixation solution, 2X

Mix 25 ml of 32% (w/v) PFA stock solution (Invitrogen/Thermo Scientific, cat. no. 047377.9M) with 75 ml of DPBS (PAN Biotech, cat. no. P04‐36500) to generate an 8% (v/v) PFA solution.
This should ideally be prepared freshly before use. Proceed with Basic Protocol 2 on the same day.

### Dye stock solutions

*MitoTracker Orange CMTMROS (Invitrogen, cat. no. M7510)*: Each vial contains 50 µg of lyophilized powder. Add 117 µl DMSO to prepare a 1 mM stock solution. Briefly vortex and spin down using a tabletop centrifuge at maximum speed for 10 s. Store the solution at –20°C protected from light.
*Cell Navigator Lysosome Staining Kit NIR Fluorescence (AAT Bioquest, cat. no. 22652)*: This kit has two components. Component A contains the LysoBrite NIR lysotropic dye, delivered as a 500× ready‐to‐use stock solution in DMSO; Component B contains the live cell staining buffer. Store both components at –20°C protected from light.
*Alexa Fluor Plus 405 Phalloidin (Invitrogen cat. no. A30104)*: Each vial contains 300 U as a lyophilized powder. Add 150 µl DMSO to prepare a 66 µM stock solution. Briefly vortex and spin down using a tabletop centrifuge at maximum speed for 10 s. Store the solution at –20°C protected from light.
*Wheat germ agglutinin (WGA), Alexa Fluor 488 Conjugate (Invitrogen, cat. no. W11261)*: Each vial contains 5 mg of lyophilized powder. Add 5 ml PBS to prepare a 1 mg/ml stock solution. Store at –20°C protected from light.
*Concanavalin A, Alexa Fluor 647 Conjugate (Invitrogen, cat. no. C21421)*: Each vial contains 5 mg of lyophilized powder. Add 1 ml of 0.1 M sodium bicarbonate to each vial to prepare a 5 mg/ml stock solution. Store the solution at –20°C, protected from light, and use within 1 month.
*SYTO 14 Green Fluorescent Nucleic Acid Stain (Invitrogen, cat. no. S7576)*: Each vial contains a 5 mM ready‐to‐use stock solution in DMSO. Store the solution at –20°C protected from light.
*Hoechst 33342 (Invitrogen, cat. no. H3570)*: Each vial contains a 10 mg/ml (16.2 mM) ready‐to‐use stock solution in water. Store the solution at 4°C protected from light.

### Basal live‐cell staining buffer, 2X

Thaw LysoBrite Component B stock solution (containing the Live Cell Staining Buffer, not including dyes; part of the Cell Navigator Lysosome Staining Kit NIR Fluorescence, AAT Bioquest, cat. no. 22652) protected from light. Mix 50 ml LysoBrite Component B with 0.5 g bovine serum albumin (BSA), low endotoxin, lyophilized powder (PAN Biotech, cat. no. P06‐139350), to generate a 1% (w/v) BSA solution. Sterile filter using a 0.22‐µm‐pore‐size filter to avoid crystals, e.g., using a bottle‐top vacuum filter system with a 0.22‐µm filter (Sigma‐Aldrich, cat. no. 430769). Aliquot the solution as needed and store at −20°C.

Based on our experience, the aliquots can be stored for at least 4 weeks and up to 6 months. Although the buffer's stability was not systematically tested, we did not observe any adverse effects on staining quality when using aliquots that had been stored long term.

### Basal fixed‐cell staining buffer, 2X

Mix 100 ml Dulbecco's Balanced Salt Solution (DPBS; PAN Biotech, cat. no. P04‐36500) with 1 g bovine serum albumin (BSA), low endotoxin, lyophilized powder (PAN Biotech, cat. no. P06‐139350) to generate a 1% (w/v) BSA solution. Sterile filter the solution using a 0.22‐µm‐pore‐size filter to avoid crystals, e.g., using a bottle‐top vacuum filter system with a 0.22‐µm filter (Sigma‐Aldrich, cat. no. 430769).

Store at 4°C. *In our experience, the aliquots can be stored for up to 6 months. Although the buffer's stability was not systematically tested, we did not observe any adverse effects on staining quality when using aliquots that had been stored long term*.

### Live‐cell staining solution, 2X

Thaw LysoBrite Component A (containing the LysoBrite NIR dye) and MitoTracker stock solution (see dye stock solutions) protected from light. Briefly vortex them and spin down for 10 sec at a tabletop centrifuge at maximal speed.

To prepare 50 ml of 2× live‐cell staining solution, add 200 µl of 500× LysoBrite Component A and 50 µl of 1 mM MitoTracker stock solutions to 49.75 ml of 2× basal live‐cell staining buffer (see recipe; also see Table 6 for a summary of dye concentrations). Mix well and keep solution protected from light.

**Table 6:** Dye Concentrations used for Cell Painting Plus*^a^*

Staining solution	Dye	Abbreviation in protocol	Concentration (stock solution)	Dilution of stock solutions to prepare [2×] staining solution	Concentration in [2×] staining solution	Final dilution in well during experiment [1×]	Final concentration in well during experiment [1×]
Live‐cell dye staining solution	Cell Navigator Lysosome Staining Kit NIR Fluorescence ‐ Component A (LysoBrite NIR dye)	LysoBrite Component A	500× in DMSO	1:250	2×	1:500	1×
	Cell Navigator Lysosome Staining Kit NIR Fluorescence ‐ Component B (Live Cell Staining Buffer)	LysoBrite Component B	100% (v/v), + with 1% (w/v) BSA	Undiluted	100% (v/v), supplemented with 1% (w/v) BSA	1:2	50% (v/v), supplemented with 0.5% (w/v) BSA
	MitoTracker Orange CMTMROS	MitoTracker	1 mM in DMSO	1:1,000	1.0 µM	1:2,000	0.5 µM
First fixed dye solution	Alexa Fluor Plus 405 Phalloidin	Phalloidin	66 µM in DMSO	1:1,000	66.0 nM	1:2,000	33.0 nM
	SYTO 14 Green Fluorescent Nucleic Acid Stain	SYTO14	5 mM in DMSO	1:8,334	0.6 µM	1:16,667	0.3 µM
Second fixed dye solution	Wheat Germ Agglutinin (WGA), Alexa Fluor 488 Conjugate	WGA	1 mg/ml in PBS	1:400	2.5 µg/ml	1:800	1.3 µg/ml
	Concanavalin A, Alexa Fluor 647 Conjugate	ConA	5 mg/ml in 0.1 M sodium bicarbonate	1:250	20.0 µg/ml	1:500	10.0 µg/ml
	Hoechst 33342	Hoechst	10 mg/ml (16.2 mM) in water	1:2,000	5.0 µg/ml (8.0 µM)	1:4,000	2.5 µg/ml (4.0 µM)

^*a*^ Table modified from von Coburg et al. (2025a) as published under Creative Commons Attribution 4.0 International License (CC BY 4.0).

Proceed with Basic Protocol 2 on the same day.

Prepare the live‐cell staining solutions freshly on the day of the experiment before proceeding with Basic Protocol 2. Note that the staining and fixation solution volumes needed for the experiment depend on the number of plates to be processed and the void volume (“dead volume”) needed for the respective pipetting device. For example, for each staining solution, 15 µl/well are needed (that is, 5.76 ml per 384‐well plate). We usually calculate assuming 7‐10 ml of void volume in the reservoirs containing the staining solution for the Janus pipetting robot to ensure that pipet tips do not aspirate any air.

### Cycle 1 staining and permeabilization solution, 2X

Thaw phalloidin and SYTO14 stock solutions protected from light (see recipe for dye stock solutions). Briefly vortex dye stock solutions and spin down for 10 sec using a tabletop centrifuge at maximum speed.

To prepare 50 ml of 2× cycle 1 staining and permeabilization solution, add 50 µl of 66 µM phalloidin and 6.25 µl of 5 mM SYTO14 to 50 ml of 2× basal fixed‐cell staining buffer (see recipe). Add 0.1 ml of 100% (v/v) Triton X‐100 (Sigma, cat. no. T8787) to 49.9 ml of the staining solution to obtain a 0.2% (v/v) Triton‐X concentration. Mix well and keep solution protected from light.

Prepare solution fresh on the day of the experiment. Proceed with Basic Protocol 2 on the same day.

### Cycle 2 staining solution, 2X

Thaw WGA and ConA stock solutions (see recipe for dye stock solutions) protected from light. Briefly vortex dye stock solutions and spin down for 10 sec using a table top centrifuge at maximum speed.

To prepare 50 ml of 2× cycle 2 staining solution, add 125 µl of 1 mg/ml WGA, 200 µl of 5 mg/ml ConA, and 25 µl of 10 mg/ml Hoechst to 50 ml of 2× basal fixed‐cell staining buffer (see recipe). Mix well and keep solution protected from light.

Prepare solution fresh on the day of the experiment. Proceed with Basic Protocol 2 on the same day.

## COMMENTARY

### Critical parameters

#### Cell seeding (Basic Protocol 1/Alternate Protocol)

For basic cell cultivation before seeding of cells on plates, we recommend passaging the cells every 2‐3 days when they reach ∼80% confluency. To minimize variability between biological replicates in a screen, set up a cell bank of cryopreserved cells, use cells at the same passage numbers, and maintain identical cultivation protocols and splitting schedules across all replicates. Ideally, use cells no later than passage 5 for seeding. For seeding cells in multiwell plates, use a plate type with a clear flat bottom that is compatible with your automated microscope of choice and suitable for cell growth. Based on our experience, 384‐well PhenoPlates (Revvity Inc.) are suited for U2OS, HepG2, and MCF‐7 cells, but 384‐well Screenstar plates (Greiner Bio‐One) work best for RPTEC cells. Notably, the cell seeding density must be adjusted depending on the growth time on the plate and the duration of the screen. Due to varying cell growth and between‐lab variations, testing cell seeding conditions for the cell line of choice in pilot experiments beforehand is recommended. In our experience, cell confluency ∼80% at the time of stimulation, and imaging of ideally 2,000 to 2,500 cells per well (for solvent control wells), result in robust phenotypic profiles.

During cell seeding, equal distribution of cells across all wells of a plate is essential for reliable results. When using multichannel pipets or other automated pipetting devices for cell seeding, make sure that cells remain in a homogeneous cell suspension during the pipetting process. When using reservoirs, mix the cell suspension well before and in between pipetting steps to avoid sedimentation of cells to the bottom of the reservoir. After seeding, allow the plates to remain in the biosafety cabinet for 1 hr before transferring them to the incubator to promote a more homogeneous cell distribution.

#### Staining (Basic Protocol 2)

During the staining procedure, ensure consistent pipetting of staining solution across all wells and plates. When using an automated pipetting and plate‐handling device, we recommend testing the respective pipetting programs beforehand and visually checking that all wells are equally pipetted and that pipet tips do not aspirate air. When processing multiple plates in parallel in an automated plate‐handling pipeline, make sure to prevent contamination of live‐cell staining solution with fixation solution: Ensure that your automated pipetting pipeline first completes pipetting all plates with live‐cell staining solution before emerging tips in fixation solution when reusing the same set of tips.

For protocol steps, when liquid must be aspirated from the plate (e.g., Basic Protocol 2, step 1), we recommend using a microplate washer to ensure efficient aspiration and wash steps. Importantly, adjust the settings of the microplate washer to the plate type used, and make sure that the cell layer is not disrupted during wash and aspiration steps.

#### Image analysis (Basic Protocol 3)

When using the Harmony CPP image analysis pipeline for the first time, we recommend checking for correct cell segmentation by manually executing the respective BB, especially *Find Nuclei*, *Find Cytoplasm*, and *Find Spots* (Nucleoli) (Table 3). BB are executed by simply clicking on the respective BB in the analysis sequence. Note that BB are executed successively, one after another. The results of the respective task performed by the BB (e.g., *Find Nuclei*) will be displayed directly in the image panel, shown in the middle of the user interface (e.g., Nuclei will be highlighted in the image). Visually check that the cell regions are correctly identified. Importantly, when using channel names other than those described in Table 2, the respective channel names must be adjusted in each of the corresponding BB in the Harmony CPP image analysis pipeline.

#### Data analysis (Basic Protocol 4 and Basic Protocol 5)

The data analysis pipeline presented here was specifically designed for data generated using the Harmony CPP image analysis pipeline. Using a different Harmony CPP image analysis pipeline than the one described in Basic Protocol 3, might result in different feature naming or different output data structure. Therefore, the *CPPAnalyzer* Jupyter notebook offers several parameters that can be easily adapted by the user to account for differences in feature naming (Table 5). The parameter “Nucleoli” requests user input as to whether nucleolus‐related features are present in the dataset. The Harmony CPP image analysis pipeline described here does generate nucleolus‐related features. Therefore, the parameter should be set to “Nucleoli = TRUE”. However, when using other Harmony pipelines that do not include nucleolus features, this parameter should be set to FALSE. The parameter “Featag” requests user input for a string that is included in all feature names. This is usually defined by the “Output population” in the Harmony analysis pipeline. If the output population was named as “Cells” (as in the Harmony CPP image analysis pipeline), each feature will have the prefix “Cells”. If Featag = “Cells”, all columns with this tag in the column header will be used for data analysis. If the output population is named differently, this parameter can be adjusted accordingly.

The *CPPAnalyzer* Jupyter notebook automatically searches the input folder for the respective exported Harmony evaluation files. Therefore, the folder structure should be implemented as described in Fig. 1C. The raw data files need to contain single cell (per object) information: that is, each row in the table should represent one measurement of all features for a single cell. Analysis of CPP data using the *CPPAnalyzer* Jupyter notebook requires the three Excel sheets Table_to_exclude_wells.xlsx, Compound_layout.xlsx, and Table_planes.xlsx (see Basic Protocol 4) to be present in the input folder. In these sheets, the plate names have to match with the plate names defined during image acquisition and image analysis (see Basic Protocol 3, step 4). If these Excel sheets are named differently, the file name can be adapted (Table 5). Note that the *CPPAnalyzer* Jupyter notebook could be, in principle, used to analyze data generated by other software than Harmony, but this requires tab‐separated text files, with each line containing information of each cell. Columns representing a morphological feature need to have one common tag, so the Jupyter notebook can identify them (see parameter “Featag” above). The columns “Column” and “Row” are needed to identify the well position.

The automated *CPPManager* KNIME workflow was designed to process normalized CPP data generated with the Harmony CPP image analysis pipeline that have been normalized with the *CPPAnalyzer* Jupyter notebook (Basic Protocol 4 and Basic Protocol 5). Using a different Harmony pipeline requires adjustment of the KNIME workflow when the output data structure or names and numbers of features or channels differ. KNIME nodes that depend on specific feature or channel names are highlighted with red color in the KNIME workflow (e.g., Rule engine node, R snippet nodes for plots). In the respective nodes, the feature or channel names need to be changed according to the new names if other image analysis pipelines resulting in different feature names are used.

In general, within a single plate, treatment conditions can be randomized. However, randomization across multiple plates—that is, with subsets of compounds and corresponding concentrations being present on different plates for each replicate—would require modifying the KNIME analysis workflow used for BMC calculation, as it would disrupt the grouping node step of this workflow. Each compound plate is expected to have the same set of compounds across all four biological replicates.

### Troubleshooting

Common problems and how to solve them are summarized in Table 7.

**Table 7:** Troubleshooting Guide

Problem	Possible cause	Solution
Collagen does not dissolve completely	Collagen stock solution has coagulated.	To increase solubility, collagen may be dissolved in 0.1 N acetic acid instead of water. Do not use collagen solutions that have been stored >1 year or are expired, and always use collagen from same batch within one screening to avoid variation.
Compound does not dissolve in the compound plate; undissolved pellet remains in the well bottom	Pipetting steps or compound mixing with automated liquid handler were insufficient.	If the compound does not dissolve completely, try to pipet the well up and down with a manual pipet until the pellet dissolves.
	Compound is insoluble at the tested concentration.	Consider testing compound solubility beforehand to ensure that all tested concentrations are soluble in the solvent (DMSO) at stock concentration and in media at the respective testing concentrations.
In the Harmony image analysis sequence, the ABB *Add Channels 4i* does not work	Incorrect version of ABB.	Be sure to use the correct version of *Add Channels 4i* for your version of Harmony: • *Add Channels 4i* v1.0.7 for Harmony v4.8 or v4.9; • *Add Channels 4i* v2.0.5 for Harmony v5.2 or v5.3.
Jupyter notebook gives the error message “*folder* doesn't contain subfolders with files tagged with *objectsFiletag*”	The folder has no file with the tag *objectsFiletag*.	Check if there is a file with the tag *objectsFiletag* in the folder.
Jupyter notebook gives the error message “Some features are not in all plates”; e.g., “xxx is in yyy but not in zzz”	Not all files have the same set of features.	Check why not all files have the same set of features.
Jupyter notebook gives the error message “*expid* is not included in *tableIncludePlanes*”	There is no information about which plane to use for particular plates.	Add the missing information to the file *tableIncludePlanes*.
Jupyter notebook gives the error message “*expid* is not included in *tablewells*”	There is no information about which wells to exclude for particular plates.	Add the missing information to the file *tablewells*.
Jupyter notebook gives the error message: “All control (e.g., DMSO) wells have <100 cells for *expid*”	Wells <100 cells are exluded. When all control wells are filtered out, normalization cannot be applied.	Check why there are so few cells in the plate “expid”. Consider to remove the plate from the analysis.
Callee workflows that are called by the *CPPManager* KNIME workflow fail	Callee workflow was opened after running and saved with executed nodes.	Always reset container‐input nodes of callee workflows before running KNIME manager workflows.
Error when executing Joiner nodes in KNIME workflows	Use of different KNIME version: Joiner nodes differ between KNIME versions.	Reset Joiner node and reconfigure (possibly need to replace joiner nodes with joiner nodes from your KNIME version used).

### Statistical Analysis

Generally, statistical analysis of CPP data follows the statistical methods previously described for CP (Caicedo et al., 2017; Nyffeler et al., 2021; Nyffeler et al., 2020) and was described in detail for CPP in von Coburg et al. (2025a) and Wedler et al. (2026). The first steps comprise data cleaning and standardization/normalization and are automatically performed by the *CPPAnalyzer* Jupyter notebook (Basic Protocol 4). The data are first cleaned by column, that is, by excluding features with missing values (typically indicated as “NA”). The proportion of missing values tolerated for each feature can be defined by adjusting the parameter *propNAfea* (Table 5). We usually exclude features with ≥15% missing values (by setting *propNAfea* = 0.15). From our past screening experience, this threshold provided sufficiently clean datasets. Importantly, the features are not filtered out from the tables, but they are set to NA. Consequently they are not used in downstream analysis. Next, the data are cleaned by row, i.e., by excluding all detected objects (single cells) containing still some missing values (“NA”) in the remaining features. The proportion of missing values tolerated for each object (single cell) can be defined by adjusting the parameter *propNAcell* (Table 5). We usually set *propNAcell* = 0 to exclude all cells with any missing values.

The *CPPAnalyzer* Jupyter notebook offers different normalization approaches: The user can choose between well‐level normalization, cell normalization, and *B*‐score normalization (Table 4). Well‐level normalization is widely applied in the CP community (Bray et al., 2016; Chandrasekaran et al., 2024; Cimini et al., 2023), but cell normalization is also used (Nyffeler et al., 2020; von Coburg et al., 2025a). *B*‐score normalization was suggested before for compensation of plate position effects (Caicedo et al., 2017; Mpindi et al., 2015). When in doubt as to which normalization option is best for their dataset, we encourage readers to test the different methods and compare the results for positive and negative control compounds (see Understanding Results).

The automated *CPPManager* KNIME workflow (Basic Protocol 5) offers the option to perform BMC modeling for dose‐response data sets. The following steps are performed automatically by the *CPPManager* KNIME workflow. The BMC describes the concentration at which a phenotypic response exceeds a defined baseline activity—that is, the benchmark response (BMR) value—which is determined from the solvent control wells. Curve‐fitting is performed using the R package *tpclfit 2* (Sheffield et al., 2022), which is implemented as an R snippet node in the *CPPManager* KNIME workflow. Five different curve‐fitting models are used (cnst, hill, poly1, poly2, pow), from which the best fitting‐curve is chosen to calculate BMC for each feature (Sheffield et al., 2022). If no BMC can be calculated for a respective feature or the calculated BMC is greater than the highest tested non‐cytotoxic concentration (BMC_high_), the feature is considered inactive (von Coburg et al., 2025a). In cases where the estimated BMC is smaller than the lowest tested non‐cytotoxic concentration (BMC_low_), the BMC cannot be reliably extrapolated from the curve fitting. In these cases, the BMC can be adjusted to a modified lowest concentration (half‐log smaller concentration than the lowest tested concentration; Eqn. 1; see Basic Protocol 5, step 11).

Equation 1 (von Coburg et al., 2025a):

$$\begin{array}{ccc} & & \log10\left(\mathrm{BM}C^{\mathrm{LOW}}\right)=\;\log10(\mathrm{lowest}\\ & & \mathrm{tested}\;\mathrm{conc}.)-0.5 \end{array}$$

An optional filtering step will exclude BMCs with a hit call of ≤0.9, missing BMDU/BMDL values, or BMC value greater than the maximal nontoxic concentration (BMC_high_) from the subsequent analysis and visualization. Including this filter is highly recommended (Feshuk et al., 2023; Nyffeler et al., 2023). This BMC filtering option can be enabled or disabled by the user of the automated *CPPManager* KNIME workflow (Basic Protocol 5, step 10).

After calculating BMCs for single features, the features can be grouped into feature categories (see Supporting Information Table 3). Note slight changes to a few feature categories compared to our previously published work (von Coburg et al., 2025a; see Supporting Information Table 4). A feature category is considered active if at least 30% of the features show a response, i.e., have a valid BMC. The active CPP feature categories are then plotted in accumulation and magnitude plots, as described before (von Coburg et al., 2025a; see Understanding Results).

### Understanding Results

The *CPPAnalyzer* Jupyter notebook (Basic Protocol 4) automatically generates several diagnostic QC plots (Fig. 2). These plots enable the user of the data analysis pipeline to examine different QC aspects. For example, we recommend checking for heterogeneous nucleus area distributions, which may be an indication of segmentation artifacts. The corresponding histogram showing the nucleus area distribution per plate is shown in Fig. 2A and as *HistRawData.pdf* in the automatically generated data output folder (Supporting Information Table 1). To mitigate potential segmentation artifacts (which may result from specific treatment conditions) without changing the image analysis sequence, one could apply the Nucleus Area Filter to exclude small nuclear fragments or other cellular debris from further analysis that were erroneously segmented as primary objects. Additionally, it is advisable to visually inspect the nucleus segmentation in the Harmony CPP image analysis pipeline (Fig. 2A′).

Next, it is advisable to examine the number of cells per well (Fig. 2B) to check that a sufficient number of cells per well were imaged and included in subsequent image analysis. This further enables the investigation of potential plate position effects that can be recognized from visible patterns of cell growth differences across the plate. Corresponding boxplots (Fig. 2B′) make it possible to spot differences of cell numbers between plates of a screen. Similarly, the fluorescence signal intensity of selected channels is visualized as heatmap and boxplot (Fig. 2C and C′) to also spot possible staining‐related plate effects. This enables spotting of, for example, edge effects (see Plate MW00923062601 in Fig. 2C) or staining artifacts introduced by pipetting errors (see Plate MW00923071002 in Fig. 2C). Special attention should be paid to the solvent control wells, as they serve as the basis for normalization and may transmit any biases introduced at this stage. All plots are generated for both the unnormalized and the normalized data. This makes it possible to examine the effect of different normalization methods on the data. If any wells show unexpected differences from other wells, we recommend examining the corresponding raw images of these wells to check for possible artifacts. If, for any reason, the wells should be excluded from analysis, this can be done via the *table_to_exclude_wells.xlsx* (see Basic Protocol 4). To evaluate the reproducibility between single replicates of the screen, correlation matrices are calculated (Fig. 2D). This could be useful to identify replicates that differ unexpectedly from the other replicates of a screen.

The *CPPManager* KNIME pipeline (Basic Protocol 5) automatically generates a variety of plots and data tables that are summarized in Supporting Information Table 2, to further explore and visualize the normalized data. For example, visualization of cell count (Fig. 3A) enables identification of cytotoxic treatment conditions leading to profound reductions in cell number. Further, the normalized features are plotted as different plot types, such as barcode‐like heatmaps or violin plots, for visualization of phenotypic profiles (Fig. 3B). Examine the generated plots and ensure that positive and negative control compounds behave as expected, with negative controls showing little to no effect and positive controls displaying strong effects in the expected channels (e.g., higher *z*‐score magnitudes). If concentration‐response data have been generated, BMCs can be calculated and plotted, with positive controls showing a large number of active BMCs, whereas negative controls should show no or very few active BMCs (Fig. 3C). The BMCs of features and feature categories are visualized using accumulation and magnitude plots to support qualitative data interpretation (Fig. 3C).

### Time Considerations

The durations of each protocol step are summarized in Table 8. Before Basic Protocol 1, a period of 1‐2 weeks (depending on the cell type) for standard cultivation of cells should be scheduled to passage cells 2‐3 times. For standard cancer cell lines, such as U2OS, MCF‐7, or HepG2, one biological replicate can usually be performed within 1 week. For example, cells can be seeded on multiwell plates on day 1 and then stimulated with test compounds on day 2. Depending on the experimental setup chosen, cells can be incubated with compounds for 24‐48 h, and one can proceed with CPP staining on day 3 or 4. We usually conduct the CPP staining and imaging procedure within 2 days, performing staining cycle 1 on day 3 or 4, followed by image acquisition (imaging time depends on the number of plates and can be done overnight). On day 4 or 5, we proceed with dye elution, staining cycle 2, and final imaging. Yet, after completing staining cycle 1, flexible pause points can be taken between image acquisition and elution/staining cycle 2 because fixed cells are less time sensitive. However, we recommend proceeding with the imaging within 2 days after staining cycle 1 to ensure consistency of dye signals, as we have, for example, observed a decrease in LysoBrite intensity after 2 days (von Coburg et al., 2025a).

**Table 8:** Expected Duration of the Main Steps of the Individual Protocols

Protocol	Step	Duration
Basic Protocol 1	Seeding cells into multiwell plates	2‐3 hr
	Cultivating cells on the multiwell plate	24 hr
	Adding test and reference compounds to cells	2‐3 hr
	Incubating of cells with compounds	24‐48 hr, depending on experimental setup
Alternate Protocol	Seeding cells into multiwell plates	2‐3 hr
	Cultivating RPTEC cells on multiwell plates with regular medium exchange	10 days
	Incubating cells with compounds	24‐48 hr, depending on experimental setup
Basic Protocol 2	Staining cycle 1	2‐3 hr
	Image acquisition, imaging cycle 1	45 min to 2.5 hr/plate, depending on fields imaged per well and laser exposure time
	Elution	15 min/plate
	Staining cycle 2	1‐2 hr
	Image acquisition, imaging cycle 2	45 min to 2.5 hr/plate, depending on fields imaged per well and laser exposure time
Basic Protocol 3	Combining images from two staining cycles into one multichannel image	0.5‐1 hr/plate, depending on fields imaged per well
	Cell segmentation and feature extraction (running the Harmony CPP image analysis pipeline)	12‐48 hr/plate; highly dependent on number of images and number of cells imaged
Basic Protocol 4	Running the *CPPAnalyzer* Jupyter notebook	5‐10 min/plate
Basic Protocol 5	Running the *CPPManager* KNIME workflow	5‐10 min/plate

### Author Contributions

**Marlene Wedler**: Writing—original draft preparation; conceptualization; writing—review and editing; investigation; methodology; validation; software; data curation. **Elena von Coburg**: Conceptualization; writing—review and editing; investigation; methodology; validation; software; data curation. **Jose M. Muino**: Software; writing—review and editing. **Lars Valentin**: Software; writing—review and editing. **Nils Körber**: Writing—review and editing; software. **Sebastian Dunst**: Conceptualization; writing—review and editing; project administration; supervision; funding acquisition; methodology; data curation; validation. **Shu Liu**: Conceptualization; writing—review and editing; project administration; supervision; funding acquisition; methodology; data curation; software; validation.

### Conflict of Interest

The authors declare no competing interests. The German Federal Institute for Risk Assessment (BfR) is a scientifically independent institution within the portfolio of the German Federal Ministry of Agriculture, Food and Regional Identity (BMLEH). The authors’ freedom to design, conduct, interpret, and publish research is explicitly not compromised. The opinions expressed in this document reflect only the author's view. The European Commission is not responsible for any use that may be made of the information it contains.

## Supporting information

Supporting Information Table 1, Output files generated by the CPPAnalyzer Jupyter notebook; Table 2, Output files generated by the CPPManager KNIME workflow; and Table 4, comparison with previously published CPP analysis approach

Supporting Information Table 3: Harmony features and feature categories

## Data Availability

The data that support the protocol are openly available in the Zenodo repository at https://doi.org/10.5281/zenodo.18184315. The *CPPAnalyzer* Jupyter notebook is openly available in the Zenodo repository at https://doi.org/10.5281/zenodo.18385218 and the CPPManager KNIME workflow at https://doi.org/10.5281/zenodo.18184356.
