# Supplementary material for: Cell Painting PLUS: An Iterative Staining‐Elution Protocol for High‐Content Phenotypic Screenings
Source: Curr Protoc. 2026 Apr 27;6:e70368. doi: 10.1002/cpz1.70368 (PMC13112130; doi:10.1002/cpz1.70368)
Supplement: Supplementary file 1 — Supporting Information Table 1, Output files generated by the CPPAnalyzer Jupyter notebook; Table 2, Output files generated by the CPPManager KNIME workflow; and Table 4, comparison with previously published CPP analysis approach [file CPZ1-6-0-s002.docx]

Supplementary Table 1: List of output files and plots generated automatically by the *CPPAnalyzer* Jupyter notebook

| **File name.**  *Note that every file will have the ScreenID defined by the parameter “screened” in the Jupyter notebook as prefix in the filename.* | **File format** | **File or plot type** | **Purpose** | **Description** |
| --- | --- | --- | --- | --- |
| Normalized-date-time  (for example MW009_U2OS-NormalizedCW-2025-12-16 13:30:37.csv) | csv | table | Data file | Normalized data according to the normalization method chosen. |
| UnNormalized-date-time | csv | table | Data file | Raw data without any normalization applied, aggregated to median when using *robust=T* or mean when using *robust=F*. |
| CellReportFiltering-platename | csv | table | analysis information | Lists the number of rows (corresponding to the number of single cell measurements) and the number of features before and after filtering (according to the filter settings chosen in the Jupyter notebook) and indicates the proportion of excluded features or cells. |
| NucleoliReportFiltering-platename | csv | table | analysis information | Lists the number of rows and the number of features before and after filtering (according to the filter settings chosen in the Jupyter notebook). |
| platename-HistRawData | PDF | histogram | Quality control (QC) and data visualization | Plots show histogram for the nucleus area [µm2] of each single cell. For every plate analyzed, a separate plot is generated. Values bigger than 1000 are set to 1000. Red dotted lines indicate thresholds for nuclear size filter set in the Jupyter notebook. If nucleus segmentation by the image analysis pipeline was incorrect or fragmented, this can for example be seen by an additional population of fragments with very small nuclear size in the histogram. |
| Concentration-BeforeNorm-Heatmaps  Concentration-AfterNorm-Heatmaps | PDF | heatmap | QC and data visualization | Heatmap showing the compound concentration for each well as stated in the compound_layout file for every single plate analyzed. Note that Concentration-BeforeNorm-Heatmaps.pdf and Concentration-AfterNorm-Heatmaps.pdf are identical as no normalization is applied to the parameter “concentration”.  This plot can for example be useful to visually verify that the plate-layout is annotated as expected or to spot typos in the concentration (such as accidentally 100 µM instead of 10 µM). Grey color indicates wells that have been excluded from analysis (as defined by the user via the table_to_exclude_wells.xlsx). |
| PercentCells-BeforeNorm-Heatmaps  PercentCells-AfterNorm-Heatmaps | PDF | heatmap | QC and data visualization | Heatmap showing percent cells relative to solvent control for every well of a plate. For every plate analyzed, a separate heatmap is generated. Note that PercentCells-BeforeNorm-Heatmaps.pdf and PercentCells-AfterNorm-Heatmaps.pdf are identical. This heatmap can be useful to identify patterns or plate position effects. |
| NumberCells-BeforeNorm-Heatmaps  NumberCells-AfterNorm-Heatmaps | PDF | heatmap | QC and data visualization | Same as PercentCells-Before/AfterNorm-Heatmaps.pdf but absolute cell number per well is shown. |
| BeforeNorm-Boxplots.pdf  AfterNorm-Boxplots.pdf | PDF | boxplot | QC and data visualization | This file contains different boxplots to visualize different parameters such as nucleus area or percent cells relative to solvent control or total number of cells per well for either all wells of a plate or solvent control wells only. This can be useful to identify unexpected differences between plates or biological replicates (for example if one plate/biological replicate has less cells than other plates). Other boxplots show median intensity for all plates (grouped by biological replicate or by row/column) to identify unexpected changes in fluorescence intensity between plates, rows or columns.  The file BeforeNorm-Boxplots.pdf shows raw data while the AfterNorm-Boxplots.pdf includes the normalized data. |
| NumberCells-AfterNorm-CorHeatmaps | PDF | Correlation heatmap | QC and data visualization | Correlation between different plates for the parameter “feacor” defined by the user in the Jupyter notebook (default: “NumberCells”). The correlation method is specified by the parameter “makeCorHeatmapMethod” in the notebook and can be either “Spearman” or “Pearson”. This plot can be useful to identify plates/ biological replicates that differ in cell number from other plates/biological replicates. |
| PercentCells-AfterNorm-CorHeatmaps | PDF | Correlation heatmap | QC and data visualization | Correlation between different plates for the parameter “Percent Cells” (relative to solvent control). The correlation method is specified by the parameter “makeCorHeatmapMethod” in the notebook and can be either “Spearman” or “Pearson.” |
| AfterNorm-CaicedoHeatmaps | PDF | Correlation heatmap | QC and data visualization | Diagnostic plot showing the correlation between plates and wells to detect batch effects as described in (Caicedo et al., 2017) |
| Intensity-featurename-median  For each intensity feature, before and after normalization,  for example:  Intensity_Nucleus_HOECHST_33342_2_Median-BeforeNorm-Heatmaps.pdf  Intensity_Nucleus_HOECHST_33342_2_Median-AfterNorm-Heatmaps.pdf | PDF | heatmap | QC and data visualization | Heatmap showing the median fluorescence intensity in the respective cellular compartment (for example nucleus) and fluorescence channel (for example Hoechst) for each well of a plate. For every plate analyzed, a separate heatmap is generated. The file BeforeNorm-Heatmaps.pdf shows raw data while the AfterNorm-Heatmaps.pdf includes the normalized data. This plot can be useful to identify plate position effects of the fluorescence intensity, for example edge or border effects.  Note that channel names from staining cycle 2 are indicated with “_2”. E.g., HOECHST_33342_2 is the channel from staining cycle 2 (with DNA stain) while “HOECHST_33342” is the corresponding channel from staining cycle 1 (with Actin stain) (see Table 2). |
| PercentReplicating.pdf | PDF | density plot | QC and data visualization | Correlation for chemicals and null distribution based on percent replicating score as described in (Way et al., 2022) |

Supplementary Table 2: List of output files and plots generated by the *CPPManage*r KNIME workflow

| **KNIME Workflow** | **Output data: Folder name** | **File name** | **File type** | **purpose** | **description** |
| --- | --- | --- | --- | --- | --- |
| 1)Cytotoxicity | Date_time_normalized_data_cytotoxicity | normalized | table | Data file | Unfiltered normalized data: z-score values for each feature, on single well level |
|  |  | Normalized_data_median_nontoxic | csv/table/excel | Data file | For each compound and concentration  z-score values for all normalized features. For each biological replicate, the median of technical replicates was calculated. Includes only non-toxic conditions (PercentCells > cytotoxicity threshold). |
|  |  | Normalized_data_median_cytotoxicity | csv | Data file | Classifies each treatment condition and as nontoxic or toxic (PercentCells < cytotoxicity threshold). For each biological replicate, the median cell count of technical replicates was calculated. |
|  |  | Cytotox_AnalysisDetails | csv | Metadata/ analysis information | Context information on data analysis, e.g. KNIME workflow version, data path, cytotoxicity threshold settings. |
|  |  | plot#1_Normalized_data_median_cytotoxicity | PDF | Data visualization | plot showing for each biological replicate the cell number (relative to solvent control) for all tested concentrations of the respective compound |
| 2)Z-score visalization | Date_time_z-score_visualization | plot#2_ActivityProfile_Median | svg | Data visualization | z-score data plotted into a heatmap that shows for each treatment condition a barcode-like profile. Median from technical and biological replicates is used. |
|  |  | normalized_MedianBRep | csv | Data file | z-score values for each feature and treatment condition, median from technical and biological replicates was calculated. Contains all compounds (except solvent control) of the screen/input file. |
|  |  | medianBRep | csv | Data file | z-score values for each feature and treatment condition, median from technical and biological replicates was calculated. Contains only a subset of compounds as defined in the Compound_Group file. |
|  |  | normalized | csv | Data file | z-score values for each feature, on single well level |
|  |  | Normalized_data_median_nontoxic | csv | Data file | For each compound group (defined by the user in the *Compound_Group.xlsx* file) one data file is saved that includes for each compound and concentration  z-score values for all normalized features. For each biological replicate, the median of technical replicates was calculated. Includes only non-toxic conditions (PercentCells > cytotoxicity threshold). |
|  |  | Plot#3_correlationmap | PDF | Data visualization | Parson correlation matrices calculated from z-score values. The median correlation score of all compounds, concentration and features. |
|  |  | Plot#4_ProfileSimilarity | PDF | Data visualization | shows the hierarchical clustering of phenotypic profiles based on spearman correlation of robust z-scores at feature level at max. non-toxic concentration |
|  |  | plot#5_ZscoreViolinPlot | PDF | Data visualization | alternative visualization showing the activity (distribution of z-scores of all features grouped by imaging channel) for each treatment condition (compound and concentration) |
|  |  | z-score_AnalysisDetails | csv | Metadata/ analysis information | Context information on data analysis, e.g. KNIME workflow version, data path etc. |
| 3)BMC_Calculation | Date_time_BMC | BMC_original | csv/table/excel | Data file | BMC modeling results for each feature and compound (includes all compounds / all compound groups) |
|  |  | BMC_30filter | csv/table/excel | Data file | BMC modeling results for each feature and compound. Only features from feature categories with >30% positive features are included. (includes all compounds / all compound groups) |
|  |  | BMC_AnalysisDetails | csv | Metadata/ analysis information | Context information on data analysis, e.g. KNIME workflow version, data path etc. |
| 4)BMC_Visualization |  | BMC_original | csv | Data file | For each compound subset (defined by the user in the *Compound_Group.xlsx* file) one data file is saved that includes BMC modeling results for each feature and compound |
|  |  | BMC_30filter | csv | Data file | For each compound subset (defined by the user in the *Compound_Group.xlsx* file) one data file is saved that includes only BMCs of features from feature categories with ≥ 30% positive features are included. |
|  |  | Plot#6_BMCProportion_clusterwardD2 | PDF | Data visualization | Proportion of features with active BMC among all features for each compound, within each channel, module, compartment, or their combinations. Columns corresponding to these factors are clustered using the Ward.D2 method to visualize which factors most strongly drive the clustering of different compounds. |
|  |  | Plot#7_BMCProportion_Colnoncluster | PDF | Data visualization | Same as Plot#6 with no column cluster. |
|  |  | Plot#8_BMCPvalueProportion | PDF | Data visualization | proportion of BMCs with a significant (p<0.05) response relative to the other compounds tested within each channel, module, compartment, or their combinations. |
|  |  | Plot#9_Barplot | PDF | Data visualization | bar plots for each compound tested showing the number of active BMCs for each channel |
|  |  | Plot#10_Accumulation Plots |  | Data visualization | feature category with ≥ 30% affected features, ranked according to their BMC |
|  |  | Plot#11_Magnitude Plots |  | Data visualization | for each single features its BMC and normalized magnitude (= maximal robust z-score) is displayed. |

Supplementary Table 4: Comparison with previously published CPP analysis approach

|  | **Feature or feature category name** | **Previously published KNIME workflows (von Coburg et al., 2025)** | ***CPPAnalyze*r Jupyter notebook (normalization)** | ***CPPManager* KNIME workflow (BMC modeling)** | **Notes** |
| --- | --- | --- | --- | --- | --- |
| feature | *AbsoluteCellNumber*=Cell_TotalNumbers | Normalization with robust z-score using median and MAD | Normalization with z-score using mean and SD | / | Note that features with discrete values are normalized differently |
| feature | Number of nucleoli per cell | Normalization with robust z-score using median and MAD | Normalization with z-score using mean and SD | / |  |
| features | *RelativeCellNumber (1-2 Nucleoli)*  *RelativeCellNumber (3-4 Nucleoli)*  *RelativeCellNumber (5+ Nucleoli)* | Normalization with z-score using mean and SD | Features not present | / | Note that the features *RelativeCellNumber (1-2 Nucleoli)*  *RelativeCellNumber (3-4 Nucleoli)*  *RelativeCellNumber (5+ Nucleoli)*  are not included in the CPPAnalyzer Jupyter notebook and downstream analysis |
| features | *All other features* | Normalization with robust z-score using median and MAD | With parameter robustNorm=T , robust z-score using median and MAD is calculated | / |  |
| Feature category | Generic_Membrane_Morphology | Feature category not present | / | Feature included:  *Neighbor contact area* | Two new feature categories (Generic_Membrane_Morphology and Generic_Cell_Number)  are implemented in the CPP KNIME Manager workflow |
| Feature category | Generic_Cell_Number | Feature category not present | / | Feature included:  *AbsoluteCellNumber*=Cell_TotalNumbers |  |
| Feature category | RNA_Nuclei_Number | Features included:  *RelativeCellNumber (1-2 Nucleoli)*  *RelativeCellNumber (3-4 Nucleoli)*  *RelativeCellNumber (5+ Nucleoli)* | / | Feature included:  *Number of nucleoli per cell* |  |
| Feature category | / | Features not assigned to any category:  *AbsolutCellNumber*  *Neighbor Contact Area*  *Number of nucleoli per cell* | / | All features are assigned to feature category |  |

References:

Caicedo, J. C., Cooper, S., Heigwer, F., Warchal, S., Qiu, P., Molnar, C., . . . Carpenter, A. E. (2017). Data-analysis strategies for image-based cell profiling. *Nat Methods, 14*(9), 849-863. doi:10.1038/nmeth.4397

von Coburg, E., Wedler, M., Muino, J. M., Wolff, C., Korber, N., Dunst, S., & Liu, S. (2025). Cell Painting PLUS: expanding the multiplexing capacity of Cell Painting-based phenotypic profiling using iterative staining-elution cycles. *Nat Commun, 16*(1), 3857. doi:10.1038/s41467-025-58765-8

Way, G. P., Natoli, T., Adeboye, A., Litichevskiy, L., Yang, A., Lu, X., . . . Carpenter, A. E. (2022). Morphology and gene expression profiling provide complementary information for mapping cell state. *Cell Syst, 13*(11), 911-923 e919. doi:10.1016/j.cels.2022.10.001
